# Supplementary material for: Determining the relative salience of recognised push variables on health professional decisions to leave the UK National Health Service (NHS) using the method of paired comparisons
Source: BMJ Open. 2023 Sep 12;13(8):e070016. doi: 10.1136/bmjopen-2022-070016 (PMC10514647; doi:10.1136/bmjopen-2022-070016)
Supplement: Supplementary data [file bmjopen-2022-070016supp001.pdf]

Appendix 1

Details of Push Variables ranking question

With refence to perceptions and beliefs relating to colleague behaviour, for a common set of variables (listed below).

Under the heading *'Why Do Staff Leave the NHS?' participants were presented with request::*

'We would like your views in more detail on how important the following issues are to explain why (RESPOPNDENT'S OCCUPATIONAL GROUP AUTOMATICALLY INSERTED HERE) staff leave the NHS. To make the task easier you will be presented with the issues two at a time.**'**

When each pair is presented, choose the issue that you think is the more important reason for (RESPOPNDENT'S OCCUPATIONAL GROUP AUTOMATICALLY INSERTED HERE) staff leaving the NHS.

Item set:

A. Time pressure
B. Staffing levels
C. Working hours
D. Workload (intensity of work)
E. Work/home-life balance
F. Pay
G. Mental health/stress
H. Recognition of contribution

Permutations of pairings (*N* = 28)

A&B; A&C; A&D; A&E; A&F; A&G; A&H

B&C; B&D; B&E; B&F; B&G; B&H

C&D; C&E; C&F; C&G; C&H

D&E; D&F; D&G; D&H

E&F; E&G; E&H

F&G; F&H

G&H

The data was gathered using an on-line platform hosted by YouGov. To mitigate presentation order bias, the order of pairings, as well as the order within each pairing (i.e. A&B or B&A) was randomised.
