# Supplementary material for: Determining the relative salience of recognised push variables on health professional decisions to leave the UK National Health Service (NHS) using the method of paired comparisons
Source: BMJ Open. 2023 Sep 12;13(8):e070016. doi: 10.1136/bmjopen-2022-070016 (PMC10514647; doi:10.1136/bmjopen-2022-070016)
Supplement: Supplementary data [file bmjopen-2022-070016supp006.pdf]

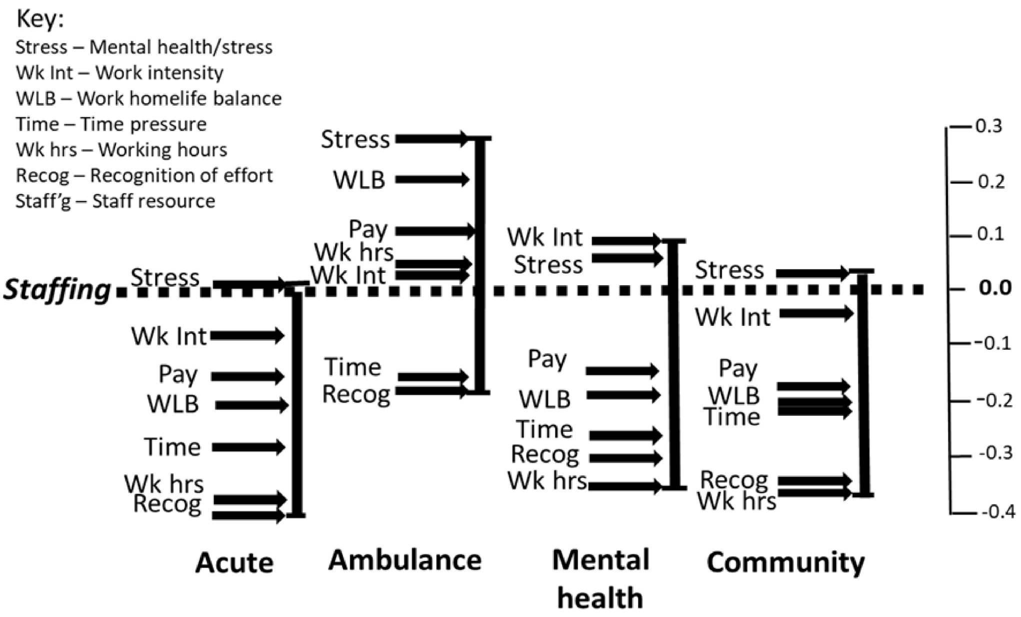

Weightings of *push* variables referenced to staffing resource level, by care provider organisation type
