# Supplementary material for: Determining the relative salience of recognised push variables on health professional decisions to leave the UK National Health Service (NHS) using the method of paired comparisons
Source: BMJ Open. 2023 Sep 12;13(8):e070016. doi: 10.1136/bmjopen-2022-070016 (PMC10514647; doi:10.1136/bmjopen-2022-070016)
Supplement: Author's manuscript [file bmjopen-2022-070016.draft_revisions.pdf]

BMJ Open

BMJ Open is committed to open peer review. As part of this commitment we make the peer review history of every article we publish publicly available.

When an article is published we post the peer reviewers' comments and the authors' responses online. We also post the versions of the paper that were used during peer review. These are the versions that the peer review comments apply to.

The versions of the paper that follow are the versions that were submitted during the peer review process. They are not the versions of record or the final published versions. They should not be cited or distributed as the published version of this manuscript.

BMJ Open is an open access journal and the full, final, typeset and author-corrected version of record of the manuscript is available on our site with no access controls, subscription charges or pay-per-view fees (http://bmjopen.bmj.com).

If you have any questions on BMJ Open's open peer review process please email info.bmjopen@bmj.com

BMJ Open

Why do health professionals leave the UK National Health Service? And what are the priorities for their retention in the Post-COVID-19 world?

Journal:	*BMJ Open*
Manuscript ID	bmjopen-2022-070016
Article Type:	Original research
Date Submitted by the Author:	17-Nov-2022

Complete List of Authors:	Weyman, Andrew; University of Bath, psychology<br>O'Hara, Rachel ; The University of Sheffield, School of Health and Related Research<br>Nolan, Peter; University of Leicester, School of Management<br>Glendinning, Richard; University of Bath, psychology<br>Coster, Joanne; The University of Sheffield<br>Roy, Deborah; University of Bath, psychology

Keywords:	Change management < HEALTH SERVICES ADMINISTRATION & MANAGEMENT, Health & safety < HEALTH SERVICES ADMINISTRATION & MANAGEMENT, HEALTH SERVICES ADMINISTRATION & MANAGEMENT, Organisation of health services < HEALTH SERVICES ADMINISTRATION & MANAGEMENT, STATISTICS & RESEARCH METHODS, OCCUPATIONAL & INDUSTRIAL MEDICINE

SCHOLARONE™
Manuscripts

I, the Submitting Author has the right to grant and does grant on behalf of all authors of the Work (as defined in the below author licence), an exclusive licence and/or a non-exclusive licence for contributions from authors who are: i) UK Crown employees; ii) where BMJ has agreed a CC-BY licence shall apply, and/or iii) in accordance with the terms applicable for US Federal Government officers or employees acting as part of their official duties; on a worldwide, perpetual, irrevocable, royalty-free basis to BMJ Publishing Group Ltd ("BMJ") its licensees and where the relevant Journal is co-owned by BMJ to the co-owners of the Journal, to publish the Work in this journal and any other BMJ products and to exploit all rights, as set out in our licence.

The Submitting Author accepts and understands that any supply made under these terms is made by BMJ to the Submitting Author unless you are acting as an employee on behalf of your employer or a postgraduate student of an affiliated institution which is paying any applicable article publishing charge ("APC") for Open Access articles. Where the Submitting Author wishes to make the Work available on an Open Access basis (and intends to pay the relevant APC), the terms of reuse of such Open Access shall be governed by a Creative Commons licence – details of these licences and which Creative Commons licence will apply to this Work are set out in our licence referred to above.

Other than as permitted in any relevant BMJ Author's Self Archiving Policies, I confirm this Work has not been accepted for publication elsewhere, is not being considered for publication elsewhere and does not duplicate material already published. I confirm all authors consent to publication of this Work and authorise the granting of this licence.

For peer review only

Why do health professionals leave the UK National Health Service? And what the priorities for their retention in the Post-COVID-19 world?

*Andrew Weyman, Reader, Department of Psychology, University of Bath, Bath, UK.

Rachel O'Hara, Senior Lecturer in Public Health, School of Health and Related Research (ScHARR), University of Sheffield, Sheffield, UK

Peter Nolan, Professor, School of Management, University of Leicester.

Richard Glendinning, Research Fellow, Department of Psychology, University of Bath.

Joanne Coster, Research Fellow, School of Health and Related Research (ScHARR), University of Sheffield, Sheffield, UK

Deborah Roy, Research Associate, Department of Psychology, University of Bath.

Corresponding author:
Dr Andrew Weyman
University of Bath
Department of Psychology
Calverton Down
Bath BA2 7AY UK
a.weyman@bath.ac.uk

Tel: 01225 385279

Word count: 4021

Keywords: Employee–retention, NHS, COVID-19, Intervention, Health profession.

ABSTRACT

Objective: Located within the context of widespread claims that primary and secondary impacts from the COVID-19 pandemic have had a detrimental impact on health professional retention, this study set out to provide human-resource staff-retention policy-relevant insight into priorities for intervention to sustain/enhance health professional (doctor, nurse, allied health and ambulance paramedic job families) retention rates by determining the relative salience of widely cited *push* influences on exit from NHS employment.

For peer review only

Design: A quantitive study to determine the relative salience of work-related stress, workload intensity, time pressure, staffing levels, working hours, work-homelife balance, recognition of effort and pay as reasons why health professionals leave NHS employment.

Setting: This is believed to be the first study that has attempted to produce a large-scale systematic assessment of drivers of exit since the emergence of the COVID-19 pandemic. During summer/autumn 2021, a UK-wide opportunity sample (*N*=2028) of NHS health professionals completed an on-line paired-comparisons exercise.

Results: Beyond the global finding highlighting the primacy of work-related stress, workload intensity and staffing levels, differences were apparent in the rank order and relative magnitude of push variables across different health professions and care delivery functions. Pay was typically located around the mid-point of the scale, while recognition of effort and working hours populated the two lowest ranks

Conclusion: Findings and implications are of relevance to informing future NHS health professional retention intervention strategy. Excepting paramedics/ambulance services, rankings of leave variables across the different health professional families and function exhibit a high degree of alignment, at the ordinal level. However, the array of demographic differences in the weightings, and detail variations in rankings, ascribed to push variables suggests that, in addition to signposting universal priorities for intervention aimed at stabilising/stemming exit rates, there may be added value from formulating bespoke, segmented, solutions for different professions and functions.

Strengths and limitations of this study

- Believed to be the first large scale systematic assessment of *push* drivers of health professional exit from NHS employment since the manifestation of the COVID-19 pandemic.
- The method of paired comparisons' produces an interval scale of the relative distance, within psychophysical space, between a set of related entities, which can also be anchored to respondent's subjective estimates of knowable, quantifiable entities, in this instance pay or staffing levels.
- Findings provide insight into the rank order and relative weighting of headline drivers of health professionals' exit from NHS employment.
- Findings are relevant to informing NHS human-resource priorities and strategy for intervention aimed at stabilising/enhancing staff retention rates.
- Findings are based on a UK wide secondary care, all-health profession opportunity sample, but with notable variability in the sizes of the occupation and care organisation type sub-samples.
- Although large for a variable ranking study of this type, the sample size was not sufficient to support multi-variate exploration of all permutations of potential interactions between the demographics and variables of interest.

For peer review only

BACKGROUND

Vacancy figures for England for the second quarter of 2022 show a record high of 132,139, a rise of 26,284 on the first quarter[1]. There are widespread claims, aligned with more substantiative evidence that the experience of working through the pandemic has significantly diminished health professional resolve/capacity to remain in NHS employment[2-4]. NHS staff survey data (2020)[5] shows a 44% increase in the proportion of staff reporting work-related stress, and around a fifth considering exiting NHS employment, both of which have been attributed to the pandemic experience[7]. There are also reports of significant numbers of staff, notably junior doctors and nursing assistants leaving to take-up better paid jobs offering better working conditions outside the NHS[6,7].

The pent-up demand for care alone has created a major imbalance between demand for treatment and the capacity to deliver[8]. Recognition of the finite scope for recruitment of migrant labour, and the inevitable time-lag in training new health professionals, combined with institutional worry over the likely magnitude of pandemic associated exits has given rise to an unprecedented focus NHS policy makers on finding ways to stabilise/enhance staff retention. This paper reports findings from a study of the relative salience and weighting of an array of widely cited *push* influences on health professional exit from NHS employment.

From the perspective of intervention aimed at enhancing retention rates, it is important to know which variables exert the strongest push, and their relative weightings. However, it is also important to determine whether this profile varies across different segments of the workforce i.e. whether 'one-size-fits all' or bespoke

For peer review only

interventions are needed[9]. Almost all previous studies of NHS staff retention are unable to answer this question, as their samples are typically be limited to single professions or institutions[10].

The study is the first large scale systematic analysis of the precursors to exit following the emergence of COVID-19. The findings are relevant to human resource intervention strategy aimed at stabilising and enhancing staff retention. They provide evidence of which variables constitute the greatest influence on employee leave decisions and the degree to which they present as universal or variable across different demographics. The focus here is on health professional migrations to non-NHS employment, as distinct from transitions from one NHS employer to another, the former representing a loss to State sector capacity, the latter producing no net change. This key distinction is frequently overlooked or blurred in reports of NHS staff turnover. The research builds on the research team's previous work within the sector[10] and broader published findings on reasons why health professional leave NHS employment.

Established insights

Pre-pandemic systems perspective research focused on the role of extrinsic aspects of work in eroding job satisfaction, mental health, physical health and strength of attachment to the NHS, notably job-demands, workload, pay, resources, and configuration of working hours[11-13]. The perspective, in essence, is a risk-based attrition model focused on the detection of precursor relationships. A high proportion of study findings point to shortage of resources, particularly staff resources as a, if not *the,* cornerstone element harbouring the potential to produce an array of

For peer review only

detrimental ripple effects. Staff shortages tend to produce time pressure, increased workload, and work rate, norming of (paid and unpaid) overtime working, with implications for employee fatigue, work-homelife balance and sickness-absence rates, mental health and burnout[14-17]. Related psychosocial effects identified include corrosive effects on morale, frustration and concern over compromised standards of patient care[18,19], amplified worry over capacity to cope, making errors and, relatedly, anxiety over personal professional vulnerability[20-23].

At a fundamental level, Issues of high/excessive workload can be conceptualised as reflecting an imbalance between rates of demand for patient care relative to resource, although other climate and culture factors can play a role, e.g. claims of increased performance monitoring, other forms of auditing and accountability related bureaucracy, have also been reported as notable sources of health professional frustration and disaffection[24,25]. Contributions from systems perspectives on mental health and work-related stress are also extensive, and principally emphasise the role of contextual effects arising from the design and configuration of work, i.e. extrinsic elements of job-demands, that embody the potential to cause psychological harms that erode staff disposition/capacity to remain[26,27].

Findings on the role of pay in exit decisions are mixed. Some claim it as a primary criterion[28,29], whereas others report it as less important than workplace climate variables, notably sufficiency of resources and staffing levels[30,31,13]. Pay is relevant in both absolute and relative terms, with linkages to notions of fairness, equity and recognition of effort[32-35]. As Dean, 2011[36] notes, dissatisfaction with pay tends to be amplified where rates remain static and are perceived by employees to be

For peer review only

disproportionate to effort expended, in the presence of significant rises in work rate and workload; this being the case since the emergence of COVID-19.

The issue of working hours, with respect to shift duration, flexibility over their configuration and the availability of part-time work has received extensive attention[37]. Findings relating to the widespread adoption of compressed hours and associated increases in typical shift duration (typically 10-12 hours) over recent decades within the NHS are mixed, liked by some staff while disliked by others[38,39,40], Irrespective of employee preferences, there are claims of long-term detrimental impacts on health, and amplified fatigue amongst older workers[37]. Increasing the availability of flexible and part-time hours is widely cited as a means of enhancing retention[39,41,42, 43].

The analysis that follows measures NHS health professionals' ratings of the relative salience of headline push influences on exit decisions after 12 months of exposure to COVID-19 working conditions.

The aim of the study was to provide future human resource policy-relevant insight into issues and priorities for intervention to sustain/enhance NHS staff retention rates.

The operational objectives were: (i) To produce on ordinal high-low ranking of headline *push* variables. (ii) To produce scaled output of the relative weighting of headline push variables. (iii) To determine the degree to which (i) and (ii) vary by health profession family, and type of secondary care organisation (acute; mental health, community and ambulance).

For peer review only

METHOD

Patient and public involvement

There was no patient involvement in this research. The project research advisory panel included health sector stakeholder representatives from NHS England/Improvement, Royal College of Nursing and UNISON.

Data collection

The data was gathered from July to December 2021, as a component of the second wave of our UK-wide survey of influences on NHS staff retention[44,45]. Ethical approval for the research was granted by University of Bath Department of Psychology Ethics Committee (reference number: PREC 20-259); and [NHS ethics] the research received National Institute for Health research (NIHR) portfolio adoption. Respondent participation was voluntary and anonymous, and all participants provided informed consent prior to taking part.

Mirroring previous research into NHS staff retention by the authors[10] the method selected for our study was paired-comparisons[46]. Paired-comparisons is well suited to eliciting views on multi-faceted subjective variables. The cognitive load on participants is low (each is requested to select one item from a randomly presented pair of items, and to repeat this for all permutations of pairings within the set). Its key strength relative to alternatives, e.g. direct ranking, subjective rating scales and sorting techniques such as Q.Sort and repertory grid, is that the output is an interval scale, that provides a quantifiable indication of the distance between the items[46,47,48], in this instance the relative salience of *push* influences.

For peer review only

(i) Generation of the item set

A set of eight widely referenced *push* variables were selected[16,49,50], six of which were common to our previous study of NHS staff retention[10]: *staffing levels*, *working hours*, *mental health/stress*, *pay*, and *recognition of contribution*. *Workload intensity* and *work-life balance* were added to the item set following consultation with government, employer, professional body and trades union stakeholders, and to take account of the prevailing pandemic working conditions, *workload intensity* and *work-life balance* were added to the item set.

(ii) Participants

Participants were a UK-wide volunteer sample recruited via the YouGov panel; 12 NHS Trusts in England and UNISON trade union members. A breakdown of the sample by health profession job-family is provided in table 1.

Table 1 – Breakdown of sample by job-family

**Profession family**	***n***
Doctors (consultant & specialists, including trainees)	227
Nurse professionals (including midwives)	687
Nurse non-professional	383
Allied health professionals	417
Ambulance Paramedics (including technicians)	243
Scientific and technical	72
**Total**	**2029**

(iii) Pre-analysis check of within respondent consistency

To consider the data set suitable for scale development, it is important to determine that participants are able to make consistent judgements, i.e. evidence that the

For peer review only

stimulus items produced logically inconsistent output, of the type A>B>C>A, would suggest that the item set does not scale. Therefore, tests of within-respondent consistency (Kendall's *K*) were performed on a pilot sample of response sets (*n* = 50). This revealed that 94% of response sets exhibited high consistency (*K* =/>0.70). The item set therefore was judged to be suitable for reliable scale development[46,47].

For peer review only

ANALYSIS

The analysis took the form of an iterative complementary approach.

(i) Generation of an NHS-wide all-profession family interval scale to demine the global profile of relative salience of the push variables in the item set.

(ii) Generation of separate, dedicated, health profession family and care delivery organisation type scales, to determine the degree of homogeneity/heterogeneity in the rank orders and weightings of the set of push variables across these demographics.

(iii) Generation of push variables scales relative to pay and staffing levels, by health profession family and care delivery organisation type.

(iv) Formal (statistical) testing of the degree of variability in the collective strength of push variables relative to pay and staffing level across different demogphics, i.e. do variables within the domain of reasons to leave constitute a stronger push with some professions/organisation types than others.

Global (all NHS health professions) ranking of push variables

In the first instance, a global all-NHS (all profession/all delivery function) interval scale of the relative importance of *push* influences was produced. Judgement proportions were determined and means for each *push* were calculated. Setting the lowest ranked variable (*recognition of contribution*) to zero, and the highest (*mental health/stress*) to 100 produced the relative weightings depicted in figure 1.

Findings point to the primacy of *mental health/stress* and *staffing levels*, *pay* is ranked fourth of the eight push variables and *recognition of contribution* and *working hours occupy* the two lowest ranks. When interpreting the findings, it is important to keep in mind that they relate to a scaling of widely cited headline drivers of exit, i.e. a scaling of *important push* influences. As such, a low ranking is not synonymous with being unimportant.

[Insert figure 1 about here]

Figure 1: Relative salience of push variables – all NHS Health Professions

Having established the global profile, deeper analyses explored differences by health profession and type of care provider organisation.

Health profession (job-family) contrasts

Contrasts were explored between doctors (consultant & specialists, including trainees), nurse professionals (including midwives), nurse non-professional, allied health professionals, and ambulance paramedics (including technicians); see table 1 (scientific and technical professionals were excluded due to restricted sample size) and four types of care provision setting: acute, mental health, community and ambulance services.

For peer review only

This analysis revealed notable alignment between profession groupings but also difference in rank orders and respective weightings. Mirroring the global profile, mental health/stress; staffing levels and workload intensity dominated the top three ranks across the different professions. However, staffing level was ascribed a notably less prominent position by ambulance participants (figure 2). Formal testing (ANOVA) revealed that that contrast with the other job families was statistically significant (*f* 4.74, *p* .004).

[Insert figure 2 about here]

Figure 2 Relative salience of push variables – by Profession job-family

Working hours, recognition of effort and time pressure consistently occupied the three lowest ranks across all profession families, except ambulance services where working hours was ranked fifth and ascribed a markedly higher weighting. Pay consistently occupied the mid-range, being ranked 4th or 5th across each of the occupation groupings, although having a higher weighting amongst nursing (most notably amongst nursing support grades; job bands 1-4) and ambulance service paramedics. The most marked contrast between the profession families related to the relative weighting of work-home life balance. Again, ambulance service paramedics present as an outlier, ranking this the second strongest influence on exit with a notably higher weighting (83). There was high consistency with respect to the ranking (4th or 5th) of this variable across the other professions, although allied health gave it a higher weighting (48) than doctors (31.6), nurse/midwife professionals (35.5) and nursing support (28.7).

For peer review only

Correlation (Pearson *r*) and linear regression (R^2) analyses, comparing variable ratings for all permutations of pairings of job-family, show high alignment across all groups, with the exception of paramedics which, once again, present as having a discrete profile (Table 2). An alternative, and complementary means of comparing the degree of between group agreement is to compare the degree of alignment at the ordinal level. Formal testing of this (kappa coefficient *K*) highlighted high congruence between nursing staff (professional and non-professional) and doctors, but modest to low alignment between other groups. Confirming the regression analysis, the most marked contrast was found between paramedics and each of the other occupations (Table 2).

Table 2
Push values - correlations between job-family ratings & rank order agreement (Pearson *r*, Cohen *K*)

Job-family	*r*	R^2	*K*
Nursing: professional v Nursing: non-professional	0.985	0.970	0.75
Doctor v Nurse professional	0.977	0.955	0.75
Nurse professional v Allied health	0.976	0.955	0.25
Doctor v Nurse non-professional	0.950	0.903	0.50
Nurse non-professional v Allied health	0.948	0.898	0.50
Allied health v Doctor	0.934	0.877	0.25
Paramedic v Allied health	0.724	0.525	0.25
Nurse non-professional v Paramedic	0.707	0.499	0.375
Nurse professional v Paramedic	0.690	0.476	0.25
Paramedic v Doctor	0.573	0.328	0.25

For peer review only

Service provider organisation contrasts

The findings (figure 3) indicate high alignment by type of care provider organisation at the level of rank order, excepting ambulance services, which again present as an outlier. However, a number of contrasts are apparent with respect to the weighting, notably *work intensity* in acute hospitals and community; *time pressure* in community and *recognition of effort* in mental health settings.

[Insert figure 3 about here]

Figure 3 Relative salience of push variables – by care-provider organisation type

Correlation analyses of variable ratings for all permutations of pairings of care provider organisation show a contrast between ambulance service and the other functions. The other three functions show strong alignment over the five highest ranked variables, with Mental health and Community sharing a common order of priority (Table 3).

Table 3
Push values - correlations between type of care provider organisation ratings & rank order agreement (Pearson *r*, Cohen *K*)

**Care provider type**	***r***	***R²***	***K***
Acute v Mental health	0.985	0.970	0.50
Acute v Community	0.982	0.964	0.50
Mental Health v Community	0.970	0.942	1.0
Mental Health v Ambulance	0.618	0.382	0.375
Acute v Ambulance	0.601	0,362	0.25
Community v Ambulance	0.50	0.302	0.5

Weightings of *push* variables relative to staffing resource and pay

Reflecting Thurstone's five method[46], the analysis proceeded to scaling the push variables relative to knowable objective value anchors of staffing levels and pay. For these separate analyses, the respective anchor item (pay or staffing) was set to zero. This produced a push scale for each profession and type of care provider organisation. Formal testing of between group differences (ANOVA) afforded insight into whether the set of headline push influences relative to pay (and staffing level) exert stronger collective leverage on some professions and care provider organisation types than others.

For peer review only

Staffing levels

Table 4 gives the relative weightings by job-family. It highlights paramedics/ ambulance services as an outlier. The profession contrasts revealed a statistically significant difference between paramedics and doctors and paramedics and qualified nurses (*f*-4.29), (*p*.006) but did not exceed the .05 threshold in comparison to allied health and nursing support).

Table 4. Normalised assessment values calculated from paired-comparisons made by five NHS health profession families

Push variable	Nurse Prof Job band 5+	Nurse non-prof Job band 4-	Allied health	Paramedics	Doctors
Time pressure	-0.331	-0.328	-0.197	-0.193	-0.402
Working Hrs	-0.421	-0.413	-0.356	0.0004	-0.521
Work intensity	-0.081	-0.096	0.020	0.143	-0.159
Work-home balance	-0.257	-0.274	-0.151	0.330	-0.400
Pay	-0.197	-0.120	-0.166	0.117	-0.328
Mental health	0.0055	0.069	0.070	0.485	-0.084
Recognition	-0.425	-0.402	-0.342	-0.253	-0.589
Staffing level	0.000	0.000	0.000	0.000	0.000

Graphical representations of these findings depicting the relative distance between push variables are provided in figure 4.

[Insert figure 4 about here]

Figure 4 Weightings of *push* variables referenced to staffing resource level, by profession

Contrasts by type of care provider (table 5) showed a significant difference between ambulance and the other care provider functions, Acute (*f*-3.98), (*p* .018); Community (*p* .040) and Mental health (*p* .056).

For peer review only

Table 5. Normalised assessment values calculated from paired-comparisons made by four NHS health care provider organisation types

Push variable	Mental health	Acute	Community	Ambulance
Time pressure	-0.283	-0.323	-0.228	-0.193
Working Hrs	-0.396	-0.412	-0.411	0.0004
Work intensity	-0.090	-0.078	-0.037	0.143
Work-home balance	-0.208	-0.248	-0.225	0.330
Pay	-0.162	-0.188	-0.193	0.117
Mental health	0.066	-0.001	0.029	0.485
Recognition	-0.342	-0.413	-0.402	-0.253
Staffing level	0.000	0.000	0.000	0.000

Graphical representations of these findings depicting the relative distance between push variables are provided in figure 5.

[Insert figure 5 about here]

Figure 5 Weightings of *push* variables referenced to staffing resource level, by care provider organisation type

Pay

In the case of pay, contrasts were explored in relation to profession and (where the sample size was sufficient) by grade, in the case of nurses (professionals, versus nursing support). Table 6 gives the relative weightings by job-family.

For peer review only

Table 6. Normalised assessment values calculated from paired-comparisons made by five NHS health profession families

Push variable	Nurse Prof Job band 5+	Nurse non-prof Job band 4-	Allied health	Paramedics	Doctors
Time pressure	-0.134	-0.208	-0.031	-0.309	-0.074
Staffing level	0.197	0.119	0.166	0.116	0.328
Working Hrs	-0,244	-0.292	-0.191	-0.116	-0.193
Work intensity	0.116	0.024	0.185	0.026	0.169
Work-home balance	-0.060	-0.154	0.014	0.213	-0.071
Mental health	0.203	0.189	0.235	0.369	0.244
Recognition	-0.228	-0.283	-0.176	-0.369	-0.261
Pay	0.000	0.000	0.000	0.000	0.000

Formal testing (ANOVA) revealed no statistically significant differences (*f*- 0.35, *p*.842). A graphical representation of the relative distance between pay and the other push variables by job-family is provided in figure 6.

[Insert figure 5 about here]

Figure 6 Weightings of *push* variables referenced to pay, by profession

DISCUSSION

Determining the relative strength of headline drivers of early-exit offers the promise of informing strategic decision-making respect to ameliorative intervention both by government and NHS employers. Insights in this area are of central relevance to signposting which push effects constitute priority issues for intervention with the potential to deliver the highest impact on health professional retention and future NHS capacity. Our overarching finding is that mental health/stress; staff resources and workload show primacy in the scaling of push influences. Pay and work-

For peer review only

homelife balance occur within the mid-range of the distribution, and recognition of effort, and working hours occupy the lowest positions.

Beyond almost universal consensus over the primacy of mental health/stress, and the basal position ascribed to working hours and recognition of effort, an array of demographic contrasts is apparent in the rankings and relative weightings ascribed to push influences. The most marked contrast is between the paramedic/ambulance services profile and other occupations/functions. At the level of rank order, this manifests in the relative position of staffing and work-home life balance. Staffing was ranked sixth by paramedics, compared with first or second and work-homelife balance was ranked second, compared with fourth or fifth by each of the other occupations. Reference to the weighting ascribed to staffing by the other professions indicates that it exerts a stronger push on doctors, allied health, and nurse professionals than non-professional nurses, and paramedics. The higher salience of work-life balance to paramedics might be interpreted as reflecting differences in the regularity and duration of their working hours. Specifically emergency ambulance crew exhibit markedly more irregular shift patterns, combined with routinised over-time working. A significant proportion of their overtime is attributable to shift ‘over-run’s’, i.e. ad-hoc, unplanned and involuntary extensions to the working day/incursions into non-work time[51]. The higher weighting of working hours by paramedics compared with the other professions aligns with this conclusion.

The much lower rank ascribed to staffing resource by paramedics is perhaps the most noteworthy finding, in view of its primacy in the scales produced by each of the

For peer review only

other personnel groups/functions, as well as the strength of objective evidence of significant shortage of paramedics[52]. It is possible that the relative social isolation of paramedics has the effect of making the issue of staff shortages less cognitively-available, i.e. less visible and immediately apparent in their work environment, than would be the case in a hospital context. Accepting this, a similar profile might have been expected from community care respondents, a high proportion of whom are also subject to peripatetic lone working, but this was not apparent. On balance, it seems questionable that social isolation would be sufficient to significantly attenuate employee awareness of staff shortages, not least because of its visible secondary impacts, i.e. increased pressure to work overtime, threats to the realisation of Trust call-out targets, as well as regular high profile coverage of staff shortages within ambulance sector publications and mass media[53,54,55].

Relative to staffing, stress, work-home life balance, work-intensity, pay and working hours exert a stronger push influence amongst paramedics than the other professions, i.e. within the leave domain these variables exert a relatively stronger push for paramedics than other professions. This explanation rests upon the premise that staff shortages might be treated as constant across each of the professions and care delivery functions, a shared benchmark against which other push variables can be scaled. If this interpretation is accepted, it suggests that attention to paramedic retention represents a key priority and source of vulnerability to the NHS. The finding from our broader survey of NHS staff retention that 24% of paramedics (N= 1157)[44] reported having submitted one or more applications for a non-NHS job in the previous six months, compared with the all-staff rate of 13% (N = 9220)[45] lends weight to this conclusion. Our data does not permit a precise explanation for the

For peer review only

marked difference in the paramedic response profile. However, the evidence that the profile of precursors to exit for paramedics is discrete from the other professions and functions presents as strong.

In contrast to some contemporary media and industrial relations accounts[55,57], and some academic research findings[58], pay, as a reason to leave did not feature prominently. Ranked fourth/fifth by each of the professions, this profile mirrored our previous research on NHS staff retention[10], which concluded that dissatisfaction with pay as an incentive to leave needs to be considered with reference to opportunities for more remunerative employment elsewhere. UK Labour Force Survey data indicates that (excepting doctors and dentists and care assistants) pay rates achieved by NHS leavers are not significantly higher, and for certain professions (notably, paramedics), they are commonly lower[37,53,6]. While other variables appear to exert a stronger push than pay, this is not grounds to diminish it as a potential source of dissatisfaction in absolute terms. However, it does suggest that attention to pay alone is unlikely to fix the retention issue.

The finding that working hours was ascribed a low rank and weighting across all professional groups and functions (again excepting paramedics) mirrors its profile in our previous work on staff retention[16] and questions the emphasis it receives within contemporary policy and employer guidance publications[42,43]. Widely cited as attractive to all employees, but particularly millennials and staff with caring responsibilities, increasing the availability of part-time and flexible hours is cast as key to increasing retention rates. However, our findings suggest that its salience to leave decisions is lower than mental health, staff resource (excepting paramedics),

For peer review only

work intensity, pay, and work-homelife balance. Mirroring our conclusions on pay, this does not represent grounds to diminish the value of flexible work hours and its attractiveness to staff, but it does suggest that attention to this issue may have a modest impact in the absence of attention to more highly ranked and variables.

The findings point to the primacy of mental health/stress as the reason why staff leave. Mental health and stress are lag effects, that can be attributable to work and non-work variables, as well as intersects between the two, mediated by individual resilience. For employers, a comprehensive perspective on intervention embodies addressing root causes attributable to the configuration of work, as well as supporting individuals at times of need[59,60]. In contrast to stress, staff resources is characterisable as a lead effect. Sufficiency of resources has material impacts on job-demands, work-life balance, time pressure and working hours as well as psychosocial impacts on psychological stress and morale.

Strength and limitations

This study is believed to be the first large scale systematic assessment of *push* variables following manifestation of the COVID-19 pandemic and its secondary impacts. Our findings provide insight into the relative weighting of headline reasons why health professionals leave NHS employment. The output is relevant to determining human resource priorities for intervention to stabilise and enhance staff retention rates.

The method of paired comparisons' produces an interval scale of the relative distance, within psychophysical space, between a set of related entities, which can also be anchored to respondent's subjective estimates of knowable quantifiable

For peer review only

entities, e.g. pay or staffing level. Respondents were asked about their beliefs regarding the exit behaviour of peers rather than their own intentions. Neither feature can be considered free from some degree unquantifiable error. However, each can be regarded as a source of common error, i.e. that *cancels-out,* when comparing the output profiles across different segments of the NHS workforce, in relative terms. The findings are based on an opportunity sample. This resulted in notable variability in the sizes of the occupation and care organisation type sub-samples. In addition, although large for a variable ranking study of this type, the sample was not sufficient to support multi-variate exploration of possible interactions between the demographics of interest.

CONCLUSIONS

Excepting paramedics, rankings of leave variables across the different health professional families exhibit a high degree of alignment, at the ordinal level, and highlight the primacy of psychological stress, staff shortages and work intensity. However, the array of demographic differences in order of priority and weightings of push variables suggests that, in addition to signposting universal priorities for intervention aimed at stabilising and stemming prevailing exit rates, it is important to consider bespoke priorities/solutions for different professions and functions. Paramedics present as an outlier, exhibiting a profile that is significantly different to other health professionals. There may also be grounds for inferring that the leave variables explored exert a relatively stronger push on members of this group.

While increases in pay are transparently important to NHS staff, findings from this research suggest that enhancements in that domain alone may produce a modest

For peer review only

impact on retention. An equivalent conclusion might be drawn with respect to the current high-profile emphasis on increased access to flexible working hours as a solution within contemporary NHS staff retention guidance to employers. Both have potential to *do-good*, but there are grounds for inferring there is a risk that neither may deliver sufficient *good* to redress the high and rising exodus in the absence of attention to what present as more fundamental drivers of exit. Importantly, scope for addressing the highest ranked drivers of exit, in large degree, lies beyond the gift of NHS employers.

The findings of this paper relate to the scaling of widely cited headline drivers of exit, i.e. a scaling of important *push* influences. As such, it would be unwise to interpret low ranked variables as unimportant. A more prudent interpretation would be to regard intervention to address low ranked variables as embodying potential to contribute to a comprehensive, multi-faceted programme of activity, but unlikely to produce the necessary degree of leverage in the absence of attention to more highly ranked, more fundamental, influences of staff exit behaviour.

For peer review only

Funding statement
This research was conducted as a component of the *'Should I stay or should I go – NHS staff retention in a post COV ID-19 world. Challenges and Prospects'*. It was funded by the Economic and Social Research Council. ESRC grant reference number ES/V015389/1.

Ethics approval statement

University of Bath, Department of Psychology Ethics Committee Reference number: 21-027

Data sharing statement
Data from the study will be available for secondary analysis within the Economic and Social Research Council repository in 2023.

Competing interests statement
There are no competing or conflicts of interest.

Authors' contributions statement
AW, PN, RO and RG conceived and designed the study. AW led the data analysis and drafted the paper, within input from PH,RO and RG. DR and JC managed the process of securing necessary ethics and governance permissions to conduct the study. DR contributed to data analysis. All authors read drafts of the manuscript and approved the final version. AW is guarantor for the paper.

Acknowledgements
The authors would like to thank the interview participants. We are also grateful for the input of our project advisory group for their guidance and assisting with facilitating access to participants.

References

1 NHS Digital. NHS Vacancy Statistics (and previous NHS Vacancies Survey), 2022; https://digital.nhs.uk/data-and-information/publications/statistical/nhs-vacancies-survey. Retrieved 3/9/22

2. O'Dowd, A. (2021) *NHS staff's stress levels rose last year as covid pandemic took its* toll. BMJ 2021*;* 372: n703 https://doi.org/10.1136/bmj.n703 (12 March) Retrieved 4/9/22.

3. De Kock, J.H., Latham, H.A., Cowden, R,G., Cullen, B., Narzisi, K., Muñoz, S-A., Leslie, S.J., McNamara, N. and Boggon, A. The mental health of NHS staff during the COVID-19 pandemic: two-wave Scottish cohort study. Cambridge University Press: 07 January 2022. https://www.cambridge.org/core/journals/bjpsych-open/article/mental-health-of-nhs-staff-during-the-covid19-pandemic-twowave-scottish-cohort-study/F77D24E3A13A7C8BC89D16E3ABF637B1.

4. Oliver, D. Act on workforce gaps, or the NHS will never recover. BMJ 2022*;* 376: n3139*.* https://doi.org/10.1136/bmj.n3139 (05 January 2022)*.*

For peer review only

5. National Health Service. NHS Staff Survey 2021 staff survey data; March, 2022. https://nhsstaffsurveyresults.com/static/1f3ea5c952df62a98b90afcf3daa29ac/ST21-National-briefing.pdf Retrieved 12/10/22.

6. Wilson, H.C.P., Abrams, S., Simpkin-Begin, A. Drexit: Understanding why junior doctors leave their training programs to train overseas: An observational study of UK physicians. Health Science Reports 2021. Wiley on-line library. https://doi.org/10.1002/hsr2.419

7. Anderson M, O'Neill C, Clark JM, Street A, Woods M, Johnston-Webber C, Charlesworth A, Whyte M, Foster M, Majeed A, Pitchforth E. Securing a sustainable and fit-for-purpose UK health and care workforce. The Lancet. 2021 May 22;397(10288):1992-2011

8. Martin, G., Clarke, J. Markar, S., Carter, A.W., Mason, S., Purkayastha, S., Darzi, A., Kinross, J. How should hospitals manage the backlog of patients awaiting surgery following the COVID-19 pandemic? A demand modelling simulation case study for carotid endarterectomy 2020; https://doi.org/10.1101/2020.04.29.20085183

9. Karanika-Murray, M. & Weyman, A. Optimising workplace interventions for health and well-being. A commentary on the limitations of the public health perspective within the workplace health arena. International journal of workplace health management. 2013; 6(2) pp.104-117.

10. Weyman, A.K., Roy, D. and Nolan, P. One-way pendulum? Staff retention in the NHS: determining the relative salience of recognised drivers of early exit. International journal of workplace health management 2020; 13(1) pp.45-60.

11. Shen, J., Cox, A., & McBride, A. Factors influencing turnover and retention of midwives and consultants: a literature review. Health services management research. 2004; https://journals.sagepub.com/doi/abs/10.1258/0951484042317769 Retrieved 4/9/22

12. Fleming, G., & Taylor, B. J. An Evaluation of Day Support: A Community Rehabilitation Service? International Journal of Disability, Community & Rehabilitation, 2010; 9(1).

13. Bimpong, K.A.A., Khan, A., Slightm, R., Tolley, C.L. and Slight, S.P. Relationship between labour force satisfaction, wages and retention within the UK National Health Service: a systematic review of the literature BMJ Open 2020; 10(7). doi.org/10.1136/bmjopen-2019-034919

14. McVicar, A, Scoping the common antecedents of job stress and job satisfaction for nurses (2000–2013) using the job demands–resources model of stress. Journal of nursing management. Journal of Nursing Management 2016; 24(2); pp. E112-E136. Wiley Online Library. https://doi.org/10.1111/jonm.12326

15. Fletcher, L., Carter, M. and Lyubovnikova, J. Congruency of resources and demands and their effects on staff turnover within the English health care sector. Journal of Occupational and Organisational Psychology 2018; 91(3); pp. 688-696. Wiley Online Library. https://doi.org/10.1111/joop.12214

For peer review only

16. Sizmur S, Raleigh V. The risks to care quality and staff wellbeing of an NHS system under pressure. The King's Fund: Oxford. 2018 Jan 31:2

17. Lopez, V., Anderson, J., West, S and Cleary M. Does the COVID-19 Pandemic Further Impact Nursing Shortages? Issues in Mental Health Nursing, 2022; 43:3, 293-295, DOI: 10.1080/01612840.2021.1977875

18. Wilson C. Why stay in nursing? Nursing Management. 2006 Feb 1;12(9); 29-32.

19. Osaro, E. and Chima, N, Challenges of a negative workload and implications on morale, productivity and quality of service delivered in NHS laboratories in England. Asian Pacific Journal of Biomedicine 2021; 4(6); pp.421-429.

20. Kelliher, C. and Parry, E. Change in healthcare: the impact on NHS managers. Journal of Organizational Change Management 2015; 28(4); pp. 591-602

21. Wise, J. Survey of UK doctors highlights blame culture within the NHS. British Medical Journal BMJ 2018;362:k4001 https://www.bmj.com/content/362/bmj.k4001.full

22. Tingle, J. Embedding a just and learning culture in the NHS. British Journal of Nursing 2019; 28(15) Published Online:8 Aug 2019 https://doi.org/10.12968/bjon.2019.28.15.1028

23. Weyman, A. K. & O'Hara, R. Decision making at the front-line: the role of choice architecture in NHS paramedic judgements over patient conveyance Chapter 10 in: P. Wankhade (Ed) Critical Perspectives in Emergency Services Management. Routledge 2019.

24. Torjessen, I. NHS is "drowning in bureaucracy" and lacks good leaders, says scathing report. British Medical Journal 2021; Vol 351. doi.org/10.1136/bmj.h3901

25. Edwards N. Turning up the heat on the NHS. bmj. 2021; Oct 29;375.

26. Ravalier JM, McVicar A, Boichat C. Work Stress in NHS Employees: A Mixed-Methods Study. International Journal of Environmental Research and Public Health 2020; 17(18):6464. https://doi.org/10.3390/ijerph17186464

27. Rattray1, J.; McCallum, L,; Hul, A.; Ramsay, P.; Salisbury, P.; Scott, T,; Cole,S,; Miller, J.; and Dixon, D. Work-related stress: the impact of COVID-19 on critical care and redeployed nurses: a mixed-methods study. BMJ Open 2021; 11(7); pp.1-7.

28. Gulland, A. Many happy returns? Health Service Journal 2001; 111, 5776, pp.12-13

29. Newman, K., Maylor, U. and Chansakar, B. The nurse satisfaction, service quality and nurse retention chain": Implications for management of recruitment and retention. Journal of Management in Medicine 2022; 16(4) pp271-291.

30. Frijters, P., Shields, M., & Wheatley Price, S. Investigating the quitting decision of nurses: Panel data evidence from the British National Health service. Health

For peer review only

Economics 2006; August. Retrieved from https://onlinelibrary.wiley.com/doi/abs/10.1002/hec.1144

31. Arnold, John; Loan-Clarke, John; Coombs, Crispin; Bosley, Sara; Martin, Caroline (2006): Push and pull: why allied health professionals stay in, leave or return to the NHS - views of professionals inside and outside the NHS. Loughborough University. Report. 2006; https://hdl.handle.net/2134/2364

32. Johnson, N., & Mandredi, S. Older Workers in the nursing and midwifery profession. Will they stay or will they go? Challenges of active aging. S. Manfreidi and L. Vickers (Eds) 2016; pp 139-158. Springer; Palgrave Macmillan, London.

33. Williamson, K and Williams, K.J. Organisational justice, trust and perceptions of fairness in the implementation of agenda for change. Radiography, 2011; 17(1), pp.61-66. https://doi.org/10.1016/j.radi.2010.08.004

34. British Medical Association (BMA). Stay one step ahead of the 2015 NHSPS. 2016. https://www.bma.org.uk/advice/employment/pensions/2015-nhs-pension-scheme. Retrieved 14/9/22

35. French, L., Hanna, P., & Huckle, C. “If I die, they do not care”: U.K. National Health Service staff experiences of betrayal-based moral injury during COVID-19. Psychological Trauma: Theory, Research, Practice, and Policy 2022; *14*(3), 516–521. https://doi.org/10.1037/tra0001134

36. Dean, E. Staff discontent rises as inflation continues to devalue salaries. Nursing Standard 2011; 26(3) September.

37. Weyman. A. K., Meadows, P. & Buckingham, A. Extending working life audit of research relating to impacts on NHS employees. May. NHS Working Longer Review 2013. https://www.researchgate.net/profile/Pamela Meadows/publication/264933033_Extending_Working_Life_Audit_of_research_relating_to_impacts_on_NHS_Employees/links/53f 607060cf2888a74923fd1/Extending-Working-Life-Audit-of-research-relating-to-impacts-on-NHS-Employees.pdf Retrieved 1//9/22

38. Atkinson, C. and Hall, L. Flexible working and happiness in the NHS. Journal of Employee Relations 2011; 22(1); pp.88-105.

39. Health Education England Facing the Facts, Shaping the Future. A draft health and care workforce strategy for England to 2027, 2017; https://www.hee.nhs.uk/sites/default/files/documents/Facing%20the%20Facts%2C%20Shaping%20the%20Future%20%E2%80%93%20a%20draft%20health%20and%20care%20workforce%20strategy%20for%20England%20to%202027.pdf. Retrieved 28/8/22

40. Nightingale, J., Burton, M., Appleyard, R., Stevens, T. and Campbell, S. Retention of radiographers: A qualitative exploration of factors influencing decisions to leave or remain within the NHS. Radiography (London) 2021; 27(3); pp.795-802. doi: 10.1016/j.radi.2020.12.008. Epub 2021 Jan 8. PMID: 33423914.

For peer review only

41. NHS Employers. Improving staff retention - a guide of employers 2017. https://www.nhsemployers.org/news/2017/09/improving-staff-retention-a-guide-for-employers. Retrieved 1/9/22

42. Royal College of Nursing. Staff retention: guidance for employers. Nursing Standard 2018; 32, 25, 15-15. doi: 10.7748/ns.32.25.15.s11. https://journals.rcni.com/nursing-standard/staff-retention-guidance-for-employers-ns.32.25.15.s11 Retrieved 14/9/22

43. NHS Improvement A collection of practical resources to help NHS organisations to improve staff retention. 2018; https://improvement.nhs.uk/resources/improving-staff-retention. Retrieved 1/9/22

44. Coster J, O'Hara R, Glendinning R, Nolan P, Roy D, Weyman A. Impact of working through COVID-19 on ambulance staff resilience and intention to leave the NHS: a mixed methods study Emergency Medicine Journal 2022;39:e5. http://dx.doi.org/10.1136/emermed-2022-999.38

45. Weyman, A., O'Hara R., Glendinning R., Nolan P., Roy D., Coster J. Impacts of working through COVID-19 pandemic on NHS staff and future employment intentions. Presented at HSR UK Conference 2022, Sheffield.

46. Thurstone, L. L. The Measurement of Values, Chicago, IL: University of Chicago Press, 1959.

47. Bock, R. D., & Jones, L. V. The Measurement and Prediction of Judgement and Choice, London, Holden-Day, 1968.

48. Cromer, M. K., Seaver, D. A., Stillwell, W. G., & Gaddy, C. D. Generating human reliability estimates using expert judgement, Vol.2, Maryland: The Maxima Corp. U.S. Nuclear Regulatory Commission, Contract Report, 1984; NUREG/CR - 3688/2.

49. Johnson, J., Hall, L., Berzins, K., Baker, J., Melling, K., & Thompson, C. Mental healthcare staff well-being and burnout: A narrative review of trends, causes, implications and recommendations for future interventions. International journal of mental health nursing 2018; 27, pp.20-32.

50. Kim, M. & Windsor, C. Resilience and work-life balance in first-line nurse manager. Asian nursing research, 2015; 9(1); pp.21-27.

51. Barth, J., Greene, J.A., Goldstein, J. and Sibley, A. Adverse Health Effects Related to Shift Work Patterns and Work Schedule Tolerance in Emergency Medical Services Personnel: A Scoping Review. Cureus 2022; 1;14(4):e23730. doi: 10.7759/cureus.23730. PMID: 35509733; PMCID: PMC9060748.

52. NHS England Operational productivity and performance in English NHS Ambulance Trusts, (2019); https://www.england.nhs.uk/wp-content/uploads/2019/09/Operational_productivity_and_performance_NHS_Ambulance_Trusts_final.pdf Retrieved 8/9/22.

For peer review only

53. Quaile, A. Tackling the shortage of paramedics. Journal of paramedic practice, 2015; 4(7) April DOI 10.12968/jpar.2015.7.4.167

54. Roberts, L. Long Covid worsens staff shortages in ambulance services The Telegraph 2022; 5th January. https://www.telegraph.co.uk/news/2022/01/05/long-covid-worsens-staff-shortages-ambulance-services/ Retrieved12/10/22

55. Gordon, S. Ambulance pressures taking a significant toll on staff, says UNISON, 2022; 25th April. https://www.unison.org.uk/news/press-release/2022/04/ambulance-pressures-taking-a-significant-toll-on-staff-says-unison/ Retrieved 12/10/22

56. Gregory, A. Thousands of NHS workers may quit for better-paid jobs, ministers warned. The Guardian 2022; 23rd May. https://www.theguardian.com/society/2022/may/23/thousands-of-nhs-workers-may-quit-for-better-paid-jobs-ministers-warned Retrieved 12/10/22

57. Morris, L. Four in five NHS staff ready to quit over pay. National Health Executive 2022; 17th June. https://www.nationalhealthexecutive.com/articles/four-in-five-workers-ready-to-quit-over-low-pay-nhs-workforce. Retrieved 10/9/22.

58. Buchan, J., Seccombe, I., Gershlick, B., & Charlesworth, A. In short supply. Pay policy and nurse numbers. The Health Foundation 2017.

59. Black, C. (2008) Working for a healthier tomorrow. London TSO.

60. Health and Safety Executive (2021) Work-related stress and how to manage it. https://www.hse.gov.uk/stress/causes.htm. Accessed 8/9/22.

For peer review only

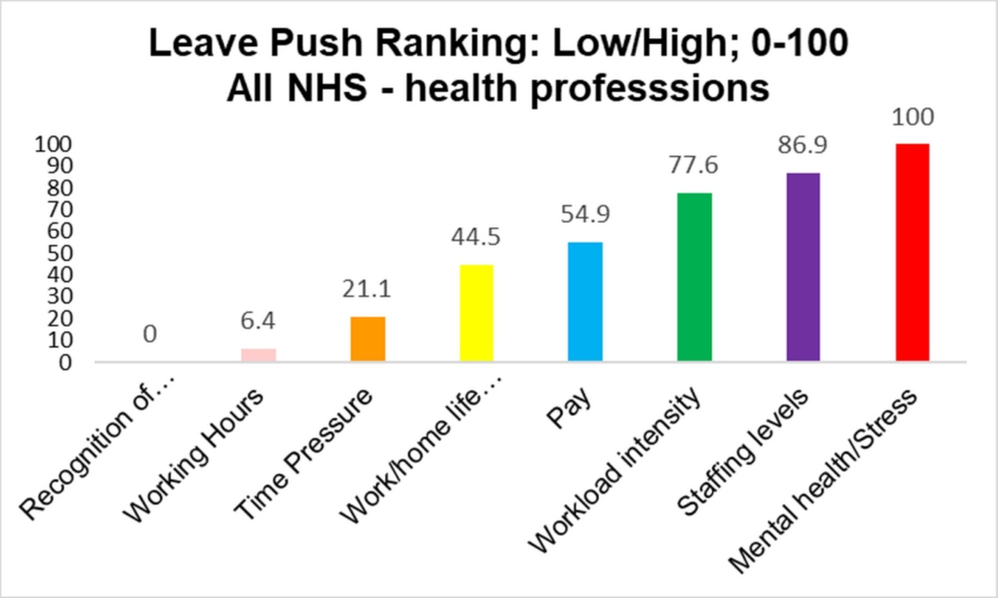

Figure 1: Relative salience of push variables – all NHS Health Professions

529x318mm (72 x 72 DPI)

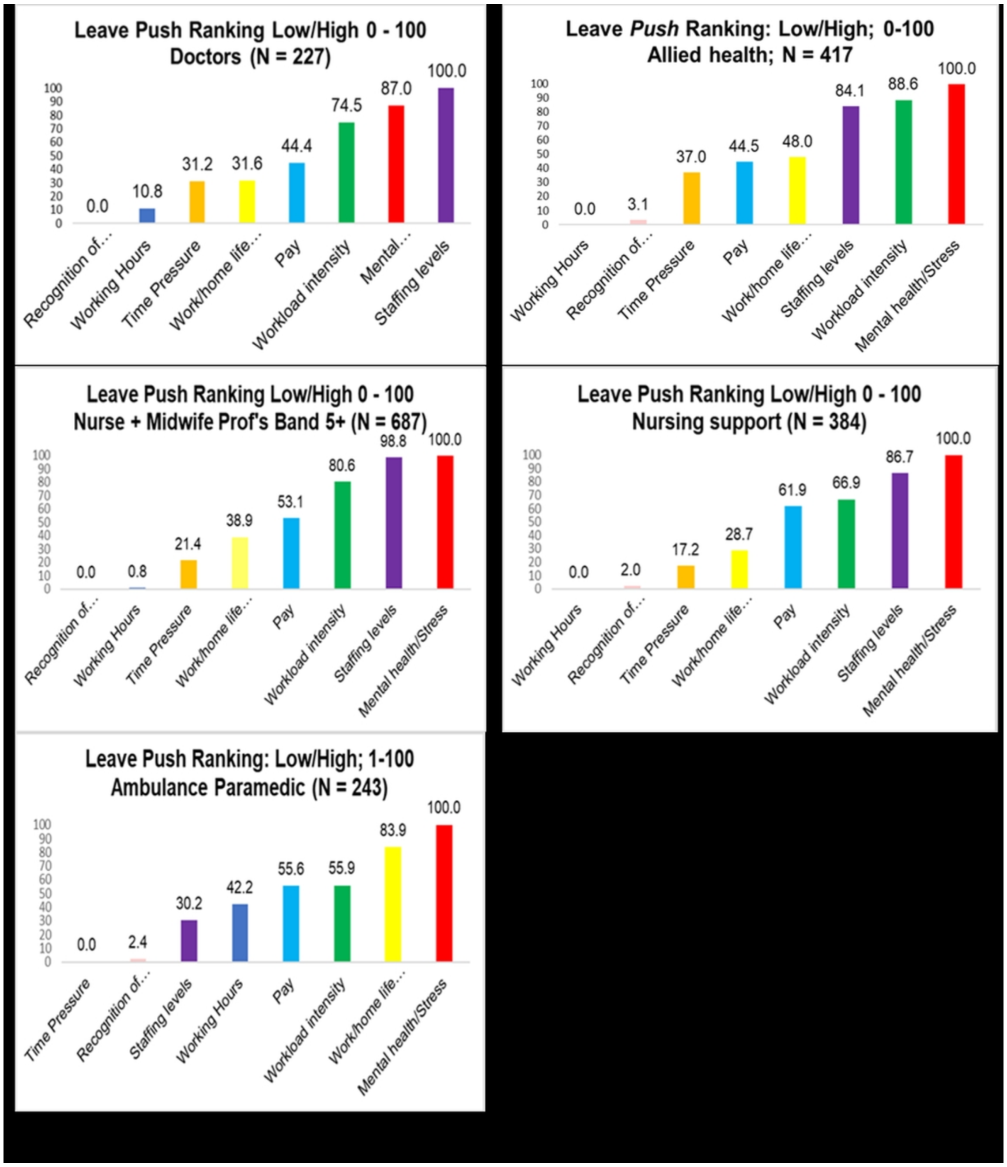

Figure 2 Relative salience of push variables – by Profession job-family

663x767mm (72 x 72 DPI)

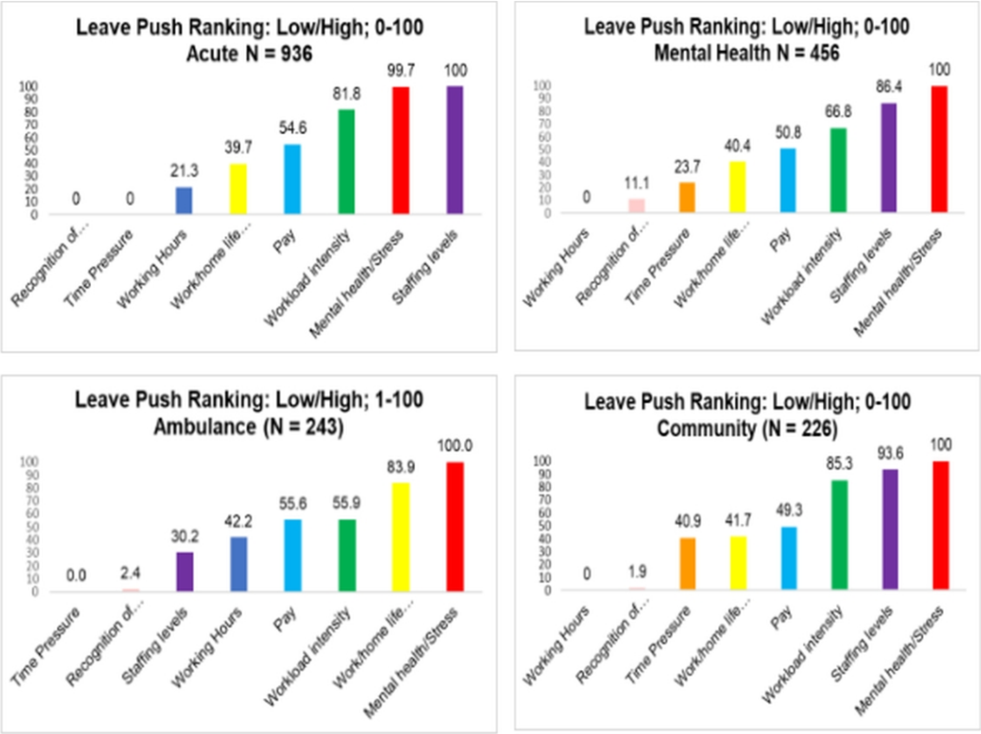

Figure 3 Relative salience of push variables – by care-provider organisation type

424x340mm (72 x 72 DPI)

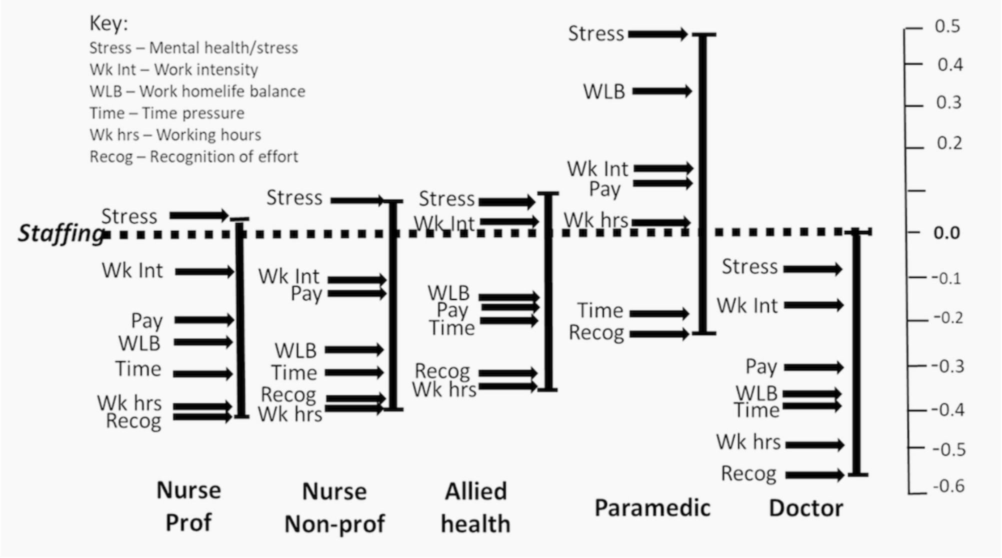

Figure 4 Weightings of push variables referenced to staffing resource level, by profession

724x403mm (72 x 72 DPI)

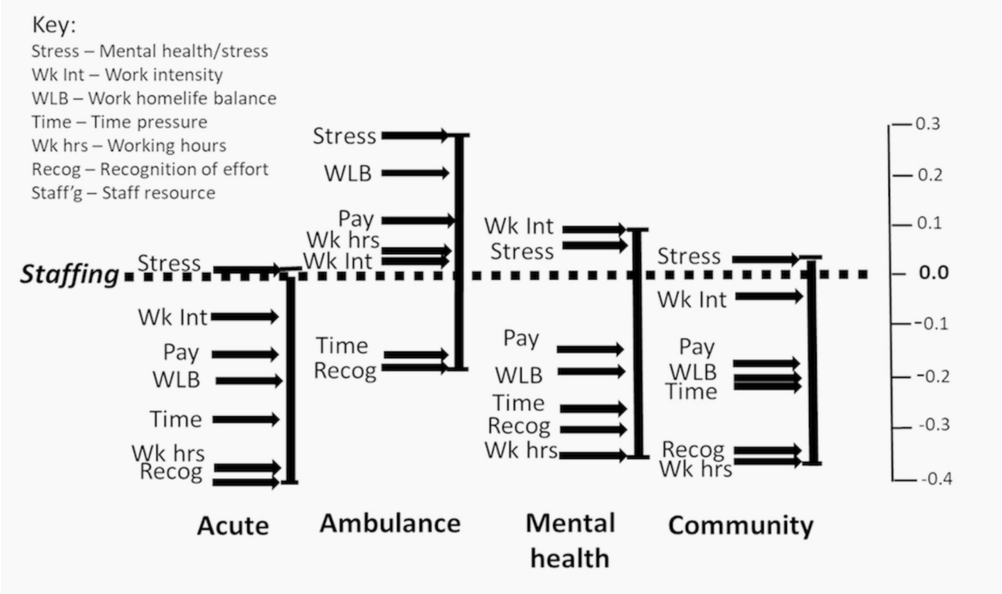

Figure 5 Weightings of push variables referenced to staffing resource level,by care provider organisation type

664x395mm (72 x 72 DPI)

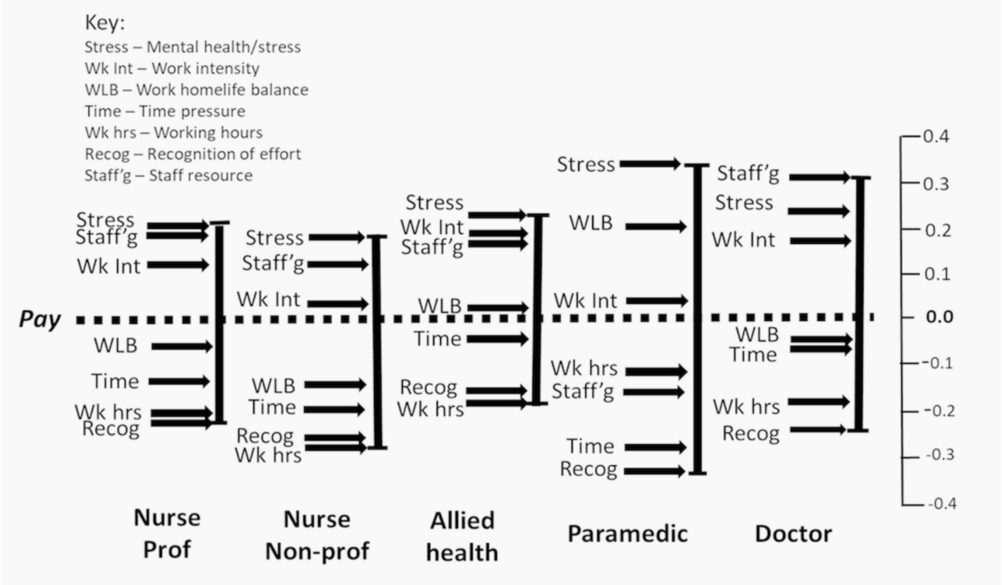

Figure 6 Weightings of push variables referenced to pay, by profession

707x413mm (72 x 72 DPI)

BMJ Open

Determining the relative salience of recognised push variables on Health Professional decisions to leave the UK National Health Service (NHS), using the method of paired-comparisons.

Journal:	*BMJ Open*
Manuscript ID	bmjopen-2022-070016.R1
Article Type:	Original research
Date Submitted by the Author:	23-May-2023
Complete List of Authors:	Weyman, Andrew; University of Bath, psychology<br>O'Hara, Rachel ; The University of Sheffield, School of Health and Related Research<br>Nolan, Peter; University of Leicester, School of Management<br>Glendinning, Richard; University of Bath, psychology<br>Coster, Joanne; The University of Sheffield<br>Roy, Deborah; University of Bath, psychology
**Primary Subject Heading**:	Health services research
Secondary Subject Heading:	Health policy
Keywords:	Change management < HEALTH SERVICES ADMINISTRATION & MANAGEMENT, Health & safety < HEALTH SERVICES ADMINISTRATION & MANAGEMENT, HEALTH SERVICES ADMINISTRATION & MANAGEMENT, Organisation of health services < HEALTH SERVICES ADMINISTRATION & MANAGEMENT, STATISTICS & RESEARCH METHODS, OCCUPATIONAL & INDUSTRIAL MEDICINE

SCHOLARONE™
Manuscripts

I, the Submitting Author has the right to grant and does grant on behalf of all authors of the Work (as defined in the below author licence), an exclusive licence and/or a non-exclusive licence for contributions from authors who are: i) UK Crown employees; ii) where BMJ has agreed a CC-BY licence shall apply, and/or iii) in accordance with the terms applicable for US Federal Government officers or employees acting as part of their official duties; on a worldwide, perpetual, irrevocable, royalty-free basis to BMJ Publishing Group Ltd ("BMJ") its licensees and where the relevant Journal is co-owned by BMJ to the co-owners of the Journal, to publish the Work in this journal and any other BMJ products and to exploit all rights, as set out in our licence.

The Submitting Author accepts and understands that any supply made under these terms is made by BMJ to the Submitting Author unless you are acting as an employee on behalf of your employer or a postgraduate student of an affiliated institution which is paying any applicable article publishing charge ("APC") for Open Access articles. Where the Submitting Author wishes to make the Work available on an Open Access basis (and intends to pay the relevant APC), the terms of reuse of such Open Access shall be governed by a Creative Commons licence – details of these licences and which Creative Commons licence will apply to this Work are set out in our licence referred to above.

Other than as permitted in any relevant BMJ Author's Self Archiving Policies, I confirm this Work has not been accepted for publication elsewhere, is not being considered for publication elsewhere and does not duplicate material already published. I confirm all authors consent to publication of this Work and authorise the granting of this licence.

For peer review only

Determining the relative salience of recognised *push* variables on Health Professional decisions to leave the UK National Health Service (NHS), using the method of paired-comparisons.

*Andrew Weyman, Reader, Department of Psychology, University of Bath, Bath, UK.

Rachel O'Hara, Senior Lecturer in Public Health, School of Health and Related Research (ScHARR), University of Sheffield, Sheffield, UK

Peter Nolan, Professor, School of Management, University of Leicester.

Richard Glendinning, Research Fellow, Department of Psychology, University of Bath.

Deborah Roy, Research Associate, Department of Psychology, University of Bath.

Joanne Coster, Research Fellow, School of Health and Related Research (ScHARR), University of Sheffield, Sheffield, UK

Corresponding author:
Dr Andrew Weyman
University of Bath
Department of Psychology
Claverton Down
Bath BA2 7AY UK
a.weyman@bath.ac.uk
Tel: 01225 385279

Word count: 4718

Keywords: Employee–retention, NHS, COVID-19, Intervention, Health professionals.

For peer review only

ABSTRACT

Objective: This study, located within the context of widespread claims that primary and secondary impacts from the COVID-19 pandemic have had a detrimental impact on health professional retention. It set out to provide staff-retention policy-relevant insight into priorities for intervention to sustain/enhance health professional (doctor, nurse, allied health and ambulance paramedic job families) retention rates by determining the relative salience of widely cited *push* influences on exit from employment in the UK National Health Service (NHS).

Design: During summer/autumn 2021, a UK-wide opportunity sample (*N*=1958) of NHS health professionals completed an on-line paired-comparisons exercise.

This quantitative study set out to determine the relative salience of work-related stress, workload intensity, time pressure, staffing levels, working hours, work-homelife balance, recognition of effort and pay as reasons why health professionals leave NHS employment.

Setting: This is believed to be the first study that has attempted to produce a large-scale systematic assessment of factors driving exit within the NHS since the emergence of the COVID-19 pandemic.

Results: Beyond the all-profession finding highlighting the primacy of work-related stress, workload intensity and staffing levels, differences were apparent in the rank order and relative magnitude of push variables across different health professions and care delivery functions. Pay was typically located around the mid-point of the scale, while recognition of effort and working hours populated the two lowest ranks

Conclusion: Findings are relevant to future NHS health professional retention intervention strategy. Excepting paramedics/ambulance services, rankings of leave variables across the different health professional families and organisation types exhibit strong alignment, at the ordinal level. However, demographic differences in the weightings, and rankings, ascribed to push factors by different professional family and organisation type suggests that, in addition to signposting universal (all-staff) priorities for intervention, there may be added value from bespoke solutions for different professions and functions.

For peer review only

Article summary

Strengths and limitations of this study

- This is a large scale systematic assessment of the relative strength of widely cited *push* factors underpinning health professional exit from NHS employment since the manifestation of the COVID-19 pandemic.
- A key strength of paired comparisons, is that the output is an interval scale, that provides a quantifiable indication of the distance between the items, in this instance the relative strength of push influences.
- Differences in the rank order and relative weighting of *push* variables between the principal NHS health profession families' are identified.
- Findings are relevant to informing NHS human-resource priorities and strategy for whole population and segmented approaches to intervention aimed at stabilising/enhancing staff retention rates.
- Although large for a variable ranking study of this type, the sample size was not sufficient to support multi-variate exploration of all permutations of potential interactions between the health profession demographics and push variables of interest.

For peer review only

BACKGROUND

NHS staff vacancy statistics for 2022, show an increase from approximately 133,100 full-time equivalent (FTE) staff in the quarter to June 2022 to around 133,400 in the quarter to September 2022. This represents a 5-year high (data are not available prior to the quarter from April – June 2017). The overall vacancy rate in the quarter to September 2022 stood at 9.7%, which also represents a 5-year high[1]. There are widespread claims, aligned with more substantiative evidence that the experience of working through the pandemic has significantly diminished health professional resolve/capacity to remain in NHS employment[2-4]. NHS staff survey data (2020)[5] shows a 44% increase in the proportion of staff reporting work-related stress, and around a fifth reporting considering exiting NHS employment, both of which have been attributed to the pandemic experience[7]. There are also reports of significant numbers of staff, notably junior doctors and nursing assistants leaving to take-up better paid jobs offering better working conditions outside the NHS[6,7]. It is important to note that the disparity between staff expressions of intention to quit and leaving behaviour can reflect systematic bias, potentially exaggerating the potential number of leavers. However, as a source of common error across successive waves of the NHS staff trend data can be considered more reliable.

The pent-up demand for care alone has created a major imbalance between demand for treatment and the capacity to deliver[8]. Recognition of the finite scope for recruitment of migrant labour, and the inevitable time-lag in training new health professionals, combined with institutional worry over the likely magnitude of pandemic associated exits has given rise to an unprecedented focus NHS policy makers on finding ways to stabilise/enhance staff retention. This paper reports

For peer review only

findings from a study of the relative salience and weighting of an array of widely cited *push* influences on health professional exit from NHS employment.

From the perspective of intervention aimed at enhancing retention rates, it is important to know which variables exert the strongest push, and their relative weightings. However, it is also important to determine whether this profile varies across different segments of the workforce i.e. whether 'one-size-fits all' or bespoke interventions are needed[9]. Almost all previous studies of NHS staff retention are unable to answer this question, as their samples are typically be limited to single professions or institutions[10].

The study is the first large scale systematic analysis of the precursors to exit following the emergence of COVID-19. The findings are relevant to human resource intervention strategy aimed at stabilising and enhancing staff retention. They provide evidence of which variables constitute the greatest influence on employee leave decisions and the degree to which they present as universal (all staff) or variable across different health profession and organisation type demographics. The focus here is on health professional migrations to non-NHS employment, as distinct from transitions from one NHS employer to another, the former representing a loss to State sector capacity, the latter producing no net change. This key distinction is frequently overlooked or blurred in reports of NHS staff turnover. The research builds on the research team's previous work within the sector[10] and broader published findings on reasons why health professional leave NHS employment.

Established insights

For peer review only

Pre-pandemic systems perspective research focused on the role of extrinsic aspects of work in eroding job satisfaction, mental health, physical health and strength of attachment to the NHS, notably job-demands, workload, pay, resources, and configuration of working hours[11-13]. The perspective, in essence, is a risk-based attrition model focused on the detection of precursor relationships. A high proportion of study findings point to shortage of resources, particularly staff resources as a, if not *the,* cornerstone element harbouring the potential to produce an array of detrimental ripple effects. Staff shortages tend to produce time pressure, increased workload, and work rate, norming of (paid and unpaid) overtime working, with implications for employee fatigue, work-homelife balance and sickness-absence rates, mental health and burnout[14-17]. Related psychosocial effects identified include corrosive effects on morale, frustration and concern over compromised standards of patient care[18,19], amplified worry over capacity to cope, making errors and, relatedly, anxiety over personal professional vulnerability[20-23].

At a fundamental level, Issues of high/excessive workload can be conceptualised as reflecting an imbalance between the demand for patient care relative to resource, although other climate and culture factors can play a role, e.g. claims of increased performance monitoring, other forms of auditing and accountability related bureaucracy, have also been reported as notable sources of health professional frustration and disaffection[24,25]. Contributions from systems perspectives on mental health and work-related stress are also extensive, and principally emphasise the role of contextual effects arising from the design and configuration of work, i.e. extrinsic elements of job-demands, that embody the potential to cause psychological harms that erode staff disposition/capacity to remain[26,27].

For peer review only

Findings on the role of pay in exit decisions are mixed. Some claim it as a primary criterion[28,29], whereas others report it as less important than workplace climate variables, notably sufficiency of resources and staffing levels[30,31,13]. Pay is relevant in both absolute and relative terms, with linkages to notions of fairness, equity and recognition of effort[32-35]. As Dean, 2011[36] notes, dissatisfaction with pay tends to be amplified where rates remain static and are perceived by employees to be disproportionate to effort expended, in the presence of significant rises in work rate and workload; this being the case since the emergence of COVID-19.

The issue of working hours, with respect to shift duration, flexibility over their configuration and the availability of part-time work has received extensive attention[37]. Findings relating to the widespread adoption of compressed hours and associated increases in typical shift duration (typically 10-12 hours) over recent decades within the NHS are mixed, liked by some staff while disliked by others[38,39,40], Irrespective of employee preferences, there are claims of long-term detrimental impacts on health, and amplified fatigue amongst older workers[37]. Increasing the availability of flexible and part-time hours is widely cited as a means of enhancing retention[39,41,42, 43].

The analysis that follows measures NHS health professionals' ratings of the relative salience of headline push influences on exit decisions after 12 months of exposure to COVID-19 working conditions.

For peer review only

The aim of the study was to provide future human resource policy-relevant insight into issues and priorities for intervention to sustain/enhance NHS staff retention rates.

The operational objectives were: (i) To produce on ordinal high-low ranking of headline *push* variables. (ii) To produce scaled output of the relative weighting of headline push variables. (iii) To determine the degree to which (i) and (ii) vary by health profession family, and type of secondary care organisation (acute; mental health, community and ambulance).

METHOD

Patient and public involvement

None. There was no patient or public involvement in this study as its focus was on NHS employees, only. Participants in the study have access to a report on headline findings from the UK-wide survey of NHS staff. The authors would like to thank members of the research stakeholder advisory group involving representatives from NHS England/Improvement, health sector professional associations and trades unions for their guidance and feedback on the content of the UK-wide survey of NHS staff of which the paired ranking exercise was a component.

Data collection

The data was gathered from July to December 2021, as a component of the second wave of the authors UK-wide on-line quantitative survey of influences on NHS staff

For peer review only

retention[44,45]. Ethical approval for the research was granted by University of Bath Department of Psychology Ethics Committee (reference number: PREC 20-259); and [NHS ethics] the research received National Institute for Health research (NIHR) portfolio adoption. Respondent participation was voluntary and anonymous, and all participants provided informed consent prior to taking part.

Mirroring previous research into NHS staff retention by the authors[10] the method selected for the study was paired-comparisons[46]. Paired-comparisons is well suited to eliciting views on multi-faceted subjective variables. The cognitive load on participants is low (each is requested to select one item from a randomly presented pair of items, and to repeat this for all permutations of pairings within the set). Its key strength relative to alternatives, e.g. direct ranking, subjective rating scales and sorting techniques such as Q.Sort and repertory grid, is that the output is an interval scale, that provides a quantifiable indication of the distance between the items[46,47,48], in this instance the relative salience of *push* influences.

(i) Generation of the item set

A set of eight widely referenced *push* variables were selected[16,49,50], six of which were common to a previous study of NHS staff retention[10]: *staffing levels*, *working hours*, *mental health/stress*, *pay*, *time pressure*, and *recognition of contribution*. *Workload intensity* and *work-life balance* were added to the item set following consultation with government, employer, professional body and trades union stakeholders, and to take account of the prevailing post-pandemic working conditions, *workload intensity* and *work-life balance* were added to the item set.

For peer review only - http://bmjopen.bmj.com/site/about/guidelines.xhtml

(ii) Reference criterion

The study set out to tap health professionals' insight into and perceptions of the relative importance of a set of widely cited reasons why colleagues within their health profession / job role leave NHS employment. Specifically, participants were asked *'how important the following issues are to explain why* (their health profession inserted) *staff leave the NHS'.*

This produced a set of subjective scales of push effects. The purpose was not to determine rates of exit, but to compare how staff beliefs about these push factors might vary between different health professional families and types of care provider organisation. The underpinning rational here was twofold. First, asking staff about their own future leave versus stay intentions was not considered a robust option as it tends to exaggerate leaving rates to an unknowable degree, due to recognised intention behaviour disparities [51], i.e. people often don't achieve their intentions. Asking participants about the behaviour of colleagues cannot be considered free from bias or error, as it is subjective, but the error can be considered common across the different groups of personnel. . Secondly, from the perspective of identifying human resource priorities for intervention aimed at stabilising/improving retention rates it is important to know the relative strength of the push factors in the item set as these can be considered to operate as precursors to exit.

(iii) Participants

Participants were a UK-wide volunteer sample recruited via the YouGov panel; UNISON trade union members and 12 NHS Trusts in England using electronic

For peer review only

mailing and newsletter communications to distribute a link to the online survey. A breakdown of the sample by health profession job-family is provided in table 1.

Table 1 Breakdown of sample by job- family

**Profession job-family**	**Numbers Employed (NHS England only)**	**Paired ranking sample**
Doctors (consultant & specialist inc trainees)[1]	124,000 (15%)	227 (12%)
Nurse professionals & Midwives[2]	332,000 (40%)	687 (35%)
Nurse non-professional[3]	280,000 (33%)	384 (20%)
Allied Health[4]	84,000 (10%)	417 (21%)
Ambulance[5]	18,000 (2%)	243 (12%)
**Total**	**837,000**	**1958**

Source: 1,2,4,5 NHS England Staff in post statistics June 2022 https://app.powerbi.com/view?r=eyJrIjoiYTlhMTAzNDEtMzQ2Ny00MDRjLWIyZTAtMTY2ZGVlZmJhMjEwIiwidCI6IjUwZjYwNzFmLWJiZmUtNDAxYS04ODAzLTY3Mzc0OGU2MjllMiIsImMiOjh9
4 Michas, F. (2022) Statista https://www.statista.com/statistics/679783/number-of-allied-health-professionals-in-nhs-workforce-england/

(iv) Procedure

Participants were presented with pairs of push factors, for all permutations of the eight factors; 28 for each participant. The order of presentation of pairs was randomised. For each pair, participants were asked *'Which of these two factors is the bigger influence on why staff in your profession / job role' leave the NHS'* (see appendix 1).

(v) Pre-analysis check of within respondent consistency

To consider the data set suitable for scale development, it is important to determine that participants are able to make consistent judgements, i.e. evidence that the stimulus items produced logically inconsistent output, of the type A>B>C>A, would suggest that the item set does not scale, and would be unsuitable for scale development. Therefore, tests of within-respondent consistency (Kendall's *K*) were performed on a pilot sample of response sets (n = 60). This revealed that 94% of

For peer review only

response sets exhibited high consistency (*K* =/>0.70). The item set therefore was judged to be suitable for reliable scale development[46,47].

ANALYSIS

The analysis took the form of an iterative complementary approach.

(i) Generation of an NHS-wide all-profession family interval scale to demine the global profile of relative salience of the push variables in the item set (see appendix 1).

(ii) Generation of separate, dedicated, health profession family and care delivery organisation type scales, to determine the degree of homogeneity/heterogeneity in the rank orders and weightings of the set of push variables between these demographics.

(iii) Generation of push variables scales relative to pay and staffing levels, by health profession family and care delivery organisation type.

(iv) Formal (statistical) testing of the degree of variability in the collective strength of push variables relative to pay and staffing level across different demographics, i.e. do variables within the domain of reasons to leave constitute a stronger push with some professions/organisation types than others.

Global (all NHS health professions) ranking of push variables

In the first instance, a global all-NHS (all profession/all delivery function) interval scale of the relative importance of *push* influences was produced. Judgement proportions were determined and means for each *push* were calculated. Setting the

For peer review only

lowest ranked variable (*recognition of contribution*) to zero, and the highest (*mental health/stress*) to 100 produced the relative weightings depicted in appendix 2.

Findings point to the primacy of *mental health/stress* and *staffing levels*, *pay* is ranked fourth of the eight push variables and *recognition of contribution* and *working hours occupy* the two lowest ranks. When interpreting the findings, it is important to keep in mind that they relate to a scaling of widely cited headline drivers of exit, i.e. a scaling of *important push* influences. As such, a low ranking is not synonymous with being unimportant.

Having established the global profile, deeper analyses explored differences by health profession and type of care provider organisation.

Health profession (job-family) contrasts

Contrasts were explored between doctors (consultant & specialists, including trainees), nurse professionals and midwives, nurse non-professional, allied health professionals, and ambulance paramedics (including technicians); see table 1 (scientific and technical professionals were excluded due to restricted sample size) and four types of care provision setting: acute, mental health, community and ambulance services.

This analysis revealed notable alignment between profession groupings but also difference in rank orders and respective weightings. Mirroring the global profile, mental health/stress; staffing levels and workload intensity dominated the top three ranks across the different professions. However, staffing level was ascribed a notably less prominent position by ambulance participants (see appendix 3). Formal

For peer review only

testing (ANOVA) revealed that that contrast with the other job families was statistically significant (F-stat 4.74, p-value .004).

Working hours, recognition of effort and time pressure consistently occupied the three lowest ranks across all profession families, except ambulance services where working hours was ranked fifth and ascribed a markedly higher weighting. Pay consistently occupied the mid-range, being ranked 4th or 5th across each of the occupation groupings, although having a higher weighting amongst nursing (most notably amongst nursing support grades; job bands 1-4) and ambulance service paramedics. The most marked contrast between the profession families related to the relative weighting of work-home life balance. Again, ambulance service paramedics present as an outlier, ranking this the second strongest influence on exit with a notably higher weighting (83). There was high consistency with respect to the ranking (4th or 5th) of this variable across the other professions, although allied health gave it a higher weighting (48) than doctors (31.6), nurse/midwife professionals (35.5) and nursing support (28.7).

Correlation (Pearson *r*) and linear regression (R^2) analyses, comparing variable ratings for all permutations of pairings of job-family, show high alignment across all groups (*r* .93 -.99; R^2 .88 -.97) with the exception of paramedics which, present as having a different profile (*r* .57-.72; R^2.44 -.53). An alternative, and complementary means of comparing the degree of between group agreement is to compare the degree of alignment at the ordinal level. Formal testing of this (kappa coefficient *K*) highlighted high congruence between nursing staff (professional and non-professional) and doctors (*K*= .75), but modest to low alignment between other groups (Nurse professional v Allied health; Doctors v Nurse non-professional, *K*= .50; Nurse professional v Allied Health v Doctors, K=.375 all other contrast *K*= .25).

For peer review only

Confirming the regression analysis, the most marked contrast was found between paramedics and each of the other occupations.

Service provider organisation contrasts

The findings indicated high alignment by type of care provider organisation at the level of rank order, excepting ambulance services, which again present as an outlier. However, a number of contrasts are apparent with respect to the weighting, notably *work intensity* in acute hospitals and community; *time pressure* in community and *recognition of effort* in mental health settings (see appendix 4).

Correlation analyses of variable ratings for all permutations of pairings of care provider organisation show a contrast between ambulance service and the other functions (Acute v Mental health; Acute v Community; Mental health v Community r=>0,9, R^2>0.90). Ambulance services v's Acute, Community and Mental health, r=0.50 -.62; R^2 .30 -.38). The other three functions show strong alignment over the five highest ranked variables, with Mental health and Community sharing a common order of priority.

Weightings of *push* variables relative to staffing resource and pay

Reflecting Thurstone's five method[46], the analysis proceeded to scaling the push variables relative to knowable objective value anchors of staffing levels and pay. For these separate analyses, the respective anchor item (pay or staffing) was set to zero. This produced a push scale for each profession and type of care provider organisation. Formal testing of between group differences (ANOVA) afforded insight

into whether the set of headline push influences relative to pay (and staffing level) exert stronger collective leverage on some professions and care provider organisation types than others.

Staffing levels

Table 2 gives the relative weightings by job-family. It highlights paramedics/ ambulance services as an outlier. The profession contrasts revealed a statistically significant difference between paramedics and doctors and paramedics and qualified nurses (F-stat 4.29), (p-value .006) but did not exceed the .05 threshold in comparison to allied health and nursing support).

Table 2. Normalised assessment values calculated from paired-comparisons made by five NHS health profession families

Push variable	Nurse Prof Job band 5+	Nurse non-prof Job band 4-	Allied health	Paramedics	Doctors
Time pressure	-0.331	-0.328	-0.197	-0.193	-0.402
Working Hrs	-0.421	-0.413	-0.356	0.0004	-0.521
Work intensity	-0.081	-0.096	0.020	0.143	-0.159
Work-home balance	-0.257	-0.274	-0.151	0.330	-0.400
Pay	-0.197	-0.120	-0.166	0.117	-0.328
Mental health	0.0055	0.069	0.070	0.485	-0.084
Recognition	-0.425	-0.402	-0.342	-0.253	-0.589
Staffing level	0.000	0.000	0.000	0.000	0.000

Graphical representations of these findings depicting the relative distance between push variables are provided in appendix 5.

For peer review only

Contrasts by type of care provider (table 3) showed a significant difference between ambulance and the other care provider functions, Acute (F-stat 3.98), (p-value .018); Community (p-value .040) and Mental health (p-value .056).

Table 3. Normalised assessment values calculated from paired-comparisons made by four NHS health care provider organisation types

Push variable	Mental health	Acute	Community	Ambulance
Time pressure	-0.283	-0.323	-0.228	-0.193
Working Hrs	-0.396	-0.412	-0.411	0.0004
Work intensity	-0.090	-0.078	-0.037	0.143
Work-home balance	-0.208	-0.248	-0.225	0.330
Pay	-0.162	-0.188	-0.193	0.117
Mental health	0.066	-0.001	0.029	0.485
Recognition	-0.342	-0.413	-0.402	-0.253
Staffing level	0.000	0.000	0.000	0.000

Graphical representations of these findings depicting the relative distance between push variables are provided in appendix 6.

Pay

In the case of pay, contrasts were explored in relation to profession and (where the sample size was sufficient) by grade, in the case of nurses (professionals, versus nursing support). Table 4 gives the relative weightings by job-family.

For peer review only

Table 4. Normalised assessment values calculated from paired-comparisons made by five NHS health profession families

Push variable	Nurse Prof Job band 5+	Nurse non-prof Job band 4-	Allied health	Paramedics	Doctors
Time pressure	-0.134	-0.208	-0.031	-0.309	-0.074
Staffing level	0.197	0.119	0.166	0.116	0.328
Working Hrs	-0,244	-0.292	-0.191	-0.116	-0.193
Work intensity	0.116	0.024	0.185	0.026	0.169
Work-home balance	-0.060	-0.154	0.014	0.213	-0.071
Mental health	0.203	0.189	0.235	0.369	0.244
Recognition	-0.228	-0.283	-0.176	-0.369	-0.261
Pay	0.000	0.000	0.000	0.000	0.000

Formal testing (ANOVA) revealed no statistically significant differences (*F*-stat 0.35, p-value .842). A graphical representation of the relative distance between pay and the other push variables by job-family is provided in figure 1.

[Insert figure 1 about here]

Figure 1 Weightings of *push* variables referenced to pay, by profession

DISCUSSION

Determining the relative strength of headline factors driving early-exit offers the promise of informing strategic decision-making respect to ameliorative intervention both by government and NHS employers. Insights in this area are of central relevance to signposting which push effects constitute priority issues for intervention with the potential to deliver the highest impact on health professional retention and future NHS capacity. The overarching finding is that mental health/stress; staff resources and workload show primacy in the scaling of push influences. Pay and

For peer review only

work-homelife balance occur within the mid-range of the distribution, and recognition of effort, and working hours occupy the lowest positions.

Beyond almost universal consensus over the primacy of mental health/stress, and the basal position ascribed to working hours and recognition of effort, a number of health profession and organisation type demographic contrasts are apparent in the rankings and relative weightings ascribed to push influences. The most marked contrast is between the paramedic/ambulance services profile and other occupations/functions. Staffing was ranked sixth by paramedics, compared with first or second and work-homelife balance was ranked second, compared with fourth or fifth by each of the other occupations. The weighting ascribed to staffing by the other professions indicates that it exerts a stronger push on doctors, allied health, and nurse professionals than non-professional nurses, and paramedics. The higher salience of work-life balance to paramedics may reflect differences in the (ir)regularity and duration of their working hours. Emergency ambulance crews have markedly more irregular shift patterns, combined with routinised over-time working. A significant proportion of their overtime is attributable to shift 'over-run's', i.e. ad-hoc, unplanned and involuntary extensions to the working day/incursions into non-work time[52]. The higher weighting of working hours by paramedics compared with the other professions aligns with this conclusion.

The much lower rank ascribed to staffing resource by paramedics is perhaps the most noteworthy finding, in view of its primacy in the scales produced by each of the other personnel groups/functions, as well as the strength of objective evidence of significant shortage of paramedics[53]. It is possible that the relative social isolation of

For peer review only

paramedics has the effect of making the issue of staff shortages less cognitively-available, i.e. less visible and immediately apparent in their work environment, than would be the case in a hospital context. Accepting this, a similar profile might have been expected from community care respondents, a high proportion of whom are also subject to peripatetic lone working, but this was not apparent. On balance, it seems questionable that social isolation would be sufficient to significantly attenuate employee awareness of staff shortages, not least because of its visible secondary impacts, i.e. increased pressure to work overtime, threats to the realisation of Trust call-out targets, as well as regular high profile coverage of staff shortages within ambulance sector publications and mass media[54,55,56].

Relative to staffing, stress, work-home life balance, work-intensity, pay and working hours exert a stronger push influence amongst paramedics than the other professions, i.e. within the leave domain these variables exert a relatively stronger push for paramedics than other professions. This explanation rests upon the premise that staff shortages can be treated as constant across each of the professions and care delivery functions, a shared benchmark against which other push variables can be scaled. If this interpretation is accepted, it suggests that attention to paramedic retention represents a key priority and source of vulnerability to the NHS. The finding from the research team's broader survey of NHS staff retention that 24% of paramedics (N= 1157)[44] reported having submitted one or more applications for a non-NHS job in the previous six months, compared with the all-staff rate of 13% (N = 9220)[45] lends weight to this conclusion. The data does not permit a precise explanation for the marked difference in the paramedic response profile. However,

For peer review only

the evidence that the profile of precursors to exit for paramedics is different from the other professions and functions presents as strong.

In contrast to some contemporary media and industrial relations accounts[55,56,57], and some academic research findings[58], pay, as a reason to leave did not feature prominently. Ranked fourth/fifth by each of the professions, this profile mirrored finding from previous research on NHS staff retention[10], which concluded that dissatisfaction with pay as an incentive to leave needs to be considered with reference to opportunities for more remunerative employment elsewhere. UK Labour Force Survey data indicates that (excepting doctors and dentists and care assistants) pay rates achieved by NHS leavers are not significantly higher, and for certain professions (notably, paramedics), they are commonly lower[37,53,6]. While other variables appear to exert a stronger push than pay, this is not grounds to diminish it as a potential source of dissatisfaction in absolute terms. However, it does suggest that attention to pay alone is unlikely to fix the retention issue.

The finding that working hours was ascribed a low rank and weighting across all professional groups and functions (again excepting paramedics) mirrors its profile in previous work on staff retention[16] and questions the emphasis it receives within contemporary policy and employer guidance publications[42,43]. Widely cited as attractive to all employees, but particularly millennials and staff with caring responsibilities, increasing the availability of part-time and flexible hours is cast as key to increasing retention rates. However, the findings suggest that its salience to leave decisions is lower than mental health, staff resource (excepting paramedics), work intensity, pay, and work-homelife balance. Mirroring the conclusions on pay,

For peer review only

this does not represent grounds to diminish the value of flexible work hours and its attractiveness to staff, but it does suggest that attention to this issue may have a modest impact in the absence of attention to more highly ranked and variables.

The findings point to the primacy of mental health/stress as the reason why staff leave. Mental health and stress are lag effects, that can be attributable to work and non-work variables, as well as intersects between the two, mediated by individual resilience. For employers, a comprehensive perspective on intervention means addressing root causes attributable to the configuration of work, as well as supporting individuals at times of need[59,60]. In contrast to stress, sufficiency of staff resources is an important lead (as well as a lag) indicator.. Sufficiency of resources has material impacts on job-demands, work-life balance, time pressure and working hours as well as psychosocial impacts on psychological stress and morale.

Strength and limitations

This study is believed to be the first large scale systematic assessment of *push* variables following manifestation of the COVID-19 pandemic and its secondary impacts. The findings provide insight into the relative weighting of headline reasons why health professionals leave NHS employment. The output is relevant to determining human resource priorities for intervention to stabilise and enhance staff retention rates.

The method of paired comparisons' produces an interval scale of the relative distance, within psychophysical space, between a set of related entities, which can also be anchored to respondent's subjective estimates of knowable quantifiable entities, e.g. pay or staffing level. Respondents were asked about their beliefs

For peer review only

regarding the exit behaviour of peers rather than their own intentions. Neither feature can be considered free from some degree unquantifiable error. However, each can be regarded as a source of common error, i.e. that *cancels-out,* when comparing the output profiles across different segments of the NHS workforce, in relative terms.

The findings reported within this paper are based upon an opportunity sample of voluntary participants with notable variability in the sizes of the occupation and care organisation type sub-samples. In common with the NHS annual staff survey, all other voluntary participation employee surveys, the potential for self-selection response bias cannot be discounted.

Proportionately, the profession job-family sub-samples show alignment with NHS contemporary staff-in post ratios for doctors, qualified nurses and midwives, but under-represent nurse non-professionals and over-represent allied health professionals and ambulance staff. However, the smallest sub-sample exceeded accepted norms regarding the minimum number of participants for scale development[61] by a factor of 10. Also, it is important to note than the scales of push factors are not subject to variability due to the sample size or influenced by variation between the proportions in realised sample and actual proportions within the NHS workforce.

Although large for a variable ranking study of this type, the sample was not sufficient to support multi-variate exploration of possible interactions between the health profession and organisation type demographics of interest.

For peer review only - http://bmjopen.bmj.com/site/about/guidelines.xhtml

CONCLUSIONS

Excepting paramedics, rankings of leave variables across the different health professional families exhibit a high degree of alignment, at the ordinal level, and highlight the primacy of psychological stress, staff shortages and work intensity. However, the presence of demographic differences in order of priority and weightings of push variables suggests that, in addition to signposting universal priorities for intervention aimed at stabilising and stemming prevailing exit rates, it is important to consider bespoke priorities/solutions for different professions and functions. Paramedics present as an outlier, exhibiting a profile that is significantly different to other health professionals. There may also be grounds for inferring that the leave variables explored exert a relatively stronger push on members of this group.

While increases in pay are transparently important to NHS staff, findings from this research suggest that enhancements in that domain alone may produce a modest impact on retention. An equivalent conclusion might be drawn with respect to the current high-profile emphasis on increased access to flexible working hours as a solution within contemporary NHS staff retention guidance to employers. Both have potential to *do-good*, but there are grounds for inferring there is a risk that neither may deliver sufficient *good* to redress the high and rising exodus in the absence of attention to what present as more fundamental factors driving exit. Importantly, scope for addressing the highest ranked factors driving exit, in large degree, lies beyond the gift of NHS employers.

For peer review only

The findings of this paper relate to the scaling of widely cited headline factors driving exit, i.e. a scaling of important *push* influences. As such, it would be unwise to interpret low ranked variables as unimportant. A more prudent interpretation would be to regard intervention to address low ranked variables as embodying potential to contribute to a comprehensive, multi-faceted programme of activity, but unlikely to produce the necessary degree of leverage in the absence of attention to more highly ranked, more fundamental, influences of staff exit behaviour.

RECOMMENDATIONS

- The insights from this study can contribute to the evidence base-for prioritising push issues and demographics for intervention aimed at stabilising / enhancing NHS health professional retention.
- NHS workface policy and planning functions could review the extent to which current staff retention strategies align with factors identified in this study.
- Further research / published evidence synthesis is needed to map the scope for intervention to address the higher ranked push factors.
- The set of push factors can be considered to constitute precursors to exit. NHS employers would benefit from a tool to benchmark and regularly (e,g, annually) monitor these variables in order to gain feedback on the effectiveness of their interventions and practices aimed at stabilising/enhancing health professional retention rates.

Funding statement
Economic and Social Research Council, No: ES/V015389/1.

For peer review only

Authors' contributions

AW, PN, RO and RG conceived and designed the study. AW led the data analysis and drafted the paper. DR and JC managed the process of securing necessary ethics and governance permissions to conduct the study. DR contributed to data analysis. All authors read drafts of the manuscript and approved the final version. AW is guarantor for the paper.

Acknowledgements

The authors would like to thank the interview participants. We are also grateful for the input of the project advisory group for their guidance and assisting with facilitating access to participants.

Competing interests

None declared - there are no competing interests for any author.

Data sharing statement

The data set can be made available to researchers

References

1 Charlesworth, A. (2022) NHS Vacancy Rates point to deepening workforce crisis The Health Foundation. https://www.health.org.uk/news-and-comment/news/nhs-vacancy-rates-point-to-deepening-workforce-crisis Retrieved 20/4/23

2. O'Dowd, A. (2021) *NHS staff's stress levels rose last year as covid pandemic took its* toll. BMJ 2021*;* 372: n703 https://doi.org/10.1136/bmj.n703 (12 March) Retrieved 4/9/22.

3. De Kock, J.H., Latham, H.A., Cowden, R,G., Cullen, B., Narzisi, K., Muñoz, S-A., Leslie, S.J., McNamara, N. and Boggon, A. The mental health of NHS staff during the COVID-19 pandemic: two-wave Scottish cohort study. Cambridge University Press: 07 January 2022. https://www.cambridge.org/core/journals/bjpsych-open/article/mental-health-of-nhs-staff-during-the-covid19-pandemic-twowave-scottish-cohort-study/F77D24E3A13A7C8BC89D16E3ABF637B1.

4. Oliver, D. Act on workforce gaps, or the NHS will never recover. BMJ 2022*;* 376: n3139*.* https://doi.org/10.1136/bmj.n3139 (05 January 2022)*.*

5. National Health Service. NHS Staff Survey 2021 staff survey data; March, 2022. https://nhsstaffsurveyresults.com/static/1f3ea5c952df62a98b90afcf3daa29ac/ST21-National-briefing.pdf Retrieved 12/10/22.

6. Wilson, H.C.P., Abrams, S., Simpkin-Begin, A. Drexit: Understanding why junior doctors leave their training programs to train overseas: An observational study of UK

physicians. Health Science Reports 2021. Wiley on-line library. https://doi.org/10.1002/hsr2.419

7. Anderson M, O'Neill C, Clark JM, Street A, Woods M, Johnston-Webber C, Charlesworth A, Whyte M, Foster M, Majeed A, Pitchforth E. Securing a sustainable and fit-for-purpose UK health and care workforce. The Lancet. 2021 May 22;397(10288):1992-2011

8. Martin, G., Clarke, J. Markar, S., Carter, A.W., Mason, S., Purkayastha, S., Darzi, A., Kinross, J. How should hospitals manage the backlog of patients awaiting surgery following the COVID-19 pandemic? A demand modelling simulation case study for carotid endarterectomy 2020; https://doi.org/10.1101/2020.04.29.20085183

9. Karanika-Murray, M. & Weyman, A. Optimising workplace interventions for health and well-being. A commentary on the limitations of the public health perspective within the workplace health arena. International journal of workplace health management. 2013; 6(2) pp.104-117.

10. Weyman, A.K., Roy, D. and Nolan, P. One-way pendulum? Staff retention in the NHS: determining the relative salience of recognised drivers of early exit. International journal of workplace health management 2020; 13(1) pp.45-60.

11. Shen, J., Cox, A., & McBride, A. Factors influencing turnover and retention of midwives and consultants: a literature review. Health services management research. 2004; https://journals.sagepub.com/doi/abs/10.1258/0951484042317769 Retrieved 4/9/22

12. Fleming, G., & Taylor, B. J. An Evaluation of Day Support: A Community Rehabilitation Service? International Journal of Disability, Community & Rehabilitation, 2010; 9(1).

13. Bimpong, K.A.A., Khan, A., Slightm, R., Tolley, C.L. and Slight, S.P. Relationship between labour force satisfaction, wages and retention within the UK National Health Service: a systematic review of the literature BMJ Open 2020; 10(7). doi.org/10.1136/bmjopen-2019-034919

14. McVicar, A, Scoping the common antecedents of job stress and job satisfaction for nurses (2000–2013) using the job demands–resources model of stress. Journal of nursing management. Journal of Nursing Management 2016; 24(2); pp. E112-E136. Wiley Online Library. https://doi.org/10.1111/jonm.12326

15. Fletcher, L., Carter, M. and Lyubovnikova, J. Congruency of resources and demands and their effects on staff turnover within the English health care sector. Journal of Occupational and Organisational Psychology 2018; 91(3); pp. 688-696. Wiley Online Library. https://doi.org/10.1111/joop.12214

16. Sizmur S, Raleigh V. The risks to care quality and staff wellbeing of an NHS system under pressure. The King's Fund: Oxford. 2018 Jan 31:2

For peer review only

17. Lopez, V., Anderson, J., West, S and Cleary M. Does the COVID-19 Pandemic Further Impact Nursing Shortages? Issues in Mental Health Nursing, 2022; 43:3, 293-295, DOI: 10.1080/01612840.2021.1977875

18. Wilson C. Why stay in nursing? Nursing Management. 2006 Feb 1;12(9); 29-32.

19. Osaro, E. and Chima, N, Challenges of a negative workload and implications on morale, productivity and quality of service delivered in NHS laboratories in England. Asian Pacific Journal of Biomedicine 2021; 4(6); pp.421-429.

20. Kelliher, C. and Parry, E. Change in healthcare: the impact on NHS managers. Journal of Organizational Change Management 2015; 28(4); pp. 591-602

21. Wise, J. Survey of UK doctors highlights blame culture within the NHS. British Medical Journal BMJ 2018;362:k4001 https://www.bmj.com/content/362/bmj.k4001.full

22. Tingle, J. Embedding a just and learning culture in the NHS. British Journal of Nursing 2019; 28(15) Published Online:8 Aug 2019 https://doi.org/10.12968/bjon.2019.28.15.1028

23. Weyman, A. K. & O'Hara, R. Decision making at the front-line: the role of choice architecture in NHS paramedic judgements over patient conveyance Chapter 10 in: P. Wankhade (Ed) Critical Perspectives in Emergency Services Management. Routledge 2019.

24. Torjessen, I. NHS is "drowning in bureaucracy" and lacks good leaders, says scathing report. British Medical Journal 2021; Vol 351. doi.org/10.1136/bmj.h3901

25. Edwards N. Turning up the heat on the NHS. bmj. 2021; Oct 29;375.

26. Ravalier JM, McVicar A, Boichat C. Work Stress in NHS Employees: A Mixed-Methods Study. International Journal of Environmental Research and Public Health 2020; 17(18):6464. https://doi.org/10.3390/ijerph17186464

27. Rattray1, J.; McCallum, L,; Hul, A.; Ramsay, P.; Salisbury, P.; Scott, T,; Cole,S,; Miller, J.; and Dixon, D. Work-related stress: the impact of COVID-19 on critical care and redeployed nurses: a mixed-methods study. BMJ Open 2021; 11(7); pp.1-7.

28. Gulland, A. Many happy returns? Health Service Journal 2001; 111, 5776, pp.12-13

29. Newman, K., Maylor, U. and Chansakar, B. The nurse satisfaction, service quality and nurse retention chain": Implications for management of recruitment and retention. Journal of Management in Medicine 2022; 16(4) pp271-291.

30. Frijters, P., Shields, M., & Wheatley Price, S. Investigating the quitting decision of nurses: Panel data evidence from the British National Health service. Health Economics 2006; August. Retrieved from https://onlinelibrary.wiley.com/doi/abs/10.1002/hec.1144

For peer review only

31. Arnold, John; Loan-Clarke, John; Coombs, Crispin; Bosley, Sara; Martin, Caroline (2006): Push and pull: why allied health professionals stay in, leave or return to the NHS - views of professionals inside and outside the NHS. Loughborough University. Report. 2006; https://hdl.handle.net/2134/2364

32. Johnson, N., & Mandredi, S. Older Workers in the nursing and midwifery profession. Will they stay or will they go? Challenges of active aging. S. Manfreidi and L. Vickers (Eds) 2016; pp 139-158. Springer; Palgrave Macmillan, London.

33. Williamson, K and Williams, K.J. Organisational justice, trust and perceptions of fairness in the implementation of agenda for change. Radiography, 2011; 17(1), pp.61-66. https://doi.org/10.1016/j.radi.2010.08.004

34. British Medical Association (BMA). Stay one step ahead of the 2015 NHSPS. 2016. https://www.bma.org.uk/advice/employment/pensions/2015-nhs-pension-scheme. Retrieved 14/9/22

35. French, L., Hanna, P., & Huckle, C. "If I die, they do not care": U.K. National Health Service staff experiences of betrayal-based moral injury during COVID-19. Psychological Trauma: Theory, Research, Practice, and Policy 2022; *14*(3), 516–521. https://doi.org/10.1037/tra0001134

36. Dean, E. Staff discontent rises as inflation continues to devalue salaries. Nursing Standard 2011; 26(3) September.

37. Weyman. A. K., Meadows, P. & Buckingham, A. Extending working life audit of research relating to impacts on NHS employees. May. NHS Working Longer Review 2013. https://www.researchgate.net/profile/Pamela Meadows/publication/264933033_Extending_Working_Life_Audit_of_research_relating_to_impacts_on_NHS_Employees/links/53f 607060cf2888a74923fd1/Extending-Working-Life-Audit-of-research-relating-to-impacts-on-NHS-Employees.pdf Retrieved 1//9/22

38. Atkinson, C. and Hall, L. Flexible working and happiness in the NHS. Journal of Employee Relations 2011; 22(1); pp.88-105.

39. Health Education England Facing the Facts, Shaping the Future. A draft health and care workforce strategy for England to 2027, 2017; https://www.hee.nhs.uk/sites/default/files/documents/Facing%20the%20Facts%2C%20Shaping%20the%20Future%20%E2%80%93%20a%20draft%20health%20and%20care%20workforce%20strategy%20for%20England%20to%202027.pdf. Retrieved 28/8/22

40. Nightingale, J., Burton, M., Appleyard, R., Stevens, T. and Campbell, S. Retention of radiographers: A qualitative exploration of factors influencing decisions to leave or remain within the NHS. Radiography (London) 2021; 27(3); pp.795-802. doi: 10.1016/j.radi.2020.12.008. Epub 2021 Jan 8. PMID: 33423914.

41. NHS Employers. Improving staff retention - a guide of employers 2017. https://www.nhsemployers.org/news/2017/09/improving-staff-retention-a-guide-for-employers. Retrieved 1/9/22

For peer review only

42. Royal College of Nursing. Staff retention: guidance for employers. Nursing Standard 2018; 32, 25, 15-15. doi: 10.7748/ns.32.25.15.s11. https://journals.rcni.com/nursing-standard/staff-retention-guidance-for-employers-ns.32.25.15.s11 Retrieved 14/9/22

43. NHS Improvement A collection of practical resources to help NHS organisations to improve staff retention. 2018; https://improvement.nhs.uk/resources/improving-staff-retention. Retrieved 1/9/22

44. Coster J, O'Hara R, Glendinning R, Nolan P, Roy D, Weyman A. Impact of working through COVID-19 on ambulance staff resilience and intention to leave the NHS: a mixed methods study Emergency Medicine Journal 2022;39:e5. http://dx.doi.org/10.1136/emermed-2022-999.38

45. Weyman, A., O'Hara R., Glendinning R., Nolan P., Roy D., Coster J. Impacts of working through COVID-19 pandemic on NHS staff and future employment intentions. Presented at HSR UK Conference 2022, Sheffield.

46. Thurstone, L. L. The Measurement of Values, Chicago, IL: University of Chicago Press, 1959.

47. Bock, R. D., & Jones, L. V. The Measurement and Prediction of Judgement and Choice, London, Holden-Day, 1968.

48. Cromer, M. K., Seaver, D. A., Stillwell, W. G., & Gaddy, C. D. Generating human reliability estimates using expert judgement, Vol.2, Maryland: The Maxima Corp. U.S. Nuclear Regulatory Commission, Contract Report, 1984; NUREG/CR - 3688/2.

49. Johnson, J., Hall, L., Berzins, K., Baker, J., Melling, K., & Thompson, C. Mental healthcare staff well-being and burnout: A narrative review of trends, causes, implications and recommendations for future interventions. International journal of mental health nursing 2018; 27, pp.20-32.

50. Kim, M. & Windsor, C. Resilience and work-life balance in first-line nurse manager. Asian nursing research, 2015; 9(1); pp.21-27.

51. Wicker, A. W. (1969). Attitudes versus actions: The relationship of verbal and overt behavioral responses to attitude objects. *Journal of Social Issues, 25*(4), 41–78. https://doi.org/10.1111/j.1540-4560.1969.tb00619.x

52. Barth, J., Greene, J.A., Goldstein, J. and Sibley, A. Adverse Health Effects Related to Shift Work Patterns and Work Schedule Tolerance in Emergency Medical Services Personnel: A Scoping Review. Cureus 2022; 1;14(4):e23730. doi: 10.7759/cureus.23730. PMID: 35509733; PMCID: PMC9060748.

53. Quaile, A. Tackling the shortage of paramedics. Journal of paramedic practice, 2015; 4(7) April DOI 10.12968/jpar.2015.7.4.167

For peer review only

54. Roberts, L. Long Covid worsens staff shortages in ambulance services The Telegraph 2022; 5th January. https://www.telegraph.co.uk/news/2022/01/05/long-covid-worsens-staff-shortages-ambulance-services/ Retrieved12/10/22

55. Gordon, S. Ambulance pressures taking a significant toll on staff, says UNISON, 2022; 25th April. https://www.unison.org.uk/news/press-release/2022/04/ambulance-pressures-taking-a-significant-toll-on-staff-says-unison/ Retrieved 12/10/22

56. Gregory, A. Thousands of NHS workers may quit for better-paid jobs, ministers warned. The Guardian 2022; 23rd May. https://www.theguardian.com/society/2022/may/23/thousands-of-nhs-workers-may-quit-for-better-paid-jobs-ministers-warned Retrieved 12/10/22

57. Morris, L. Four in five NHS staff ready to quit over pay. National Health Executive 2022; 17th June. https://www.nationalhealthexecutive.com/articles/four-in-five-workers-ready-to-quit-over-low-pay-nhs-workforce. Retrieved 10/9/22.

58. Buchan, J., Seccombe, I., Gershlick, B., & Charlesworth, A. In short supply. Pay policy and nurse numbers. The Health Foundation 2017.

59. Black, C. (2008) Working for a healthier tomorrow. London TSO.

60. Health and Safety Executive (2021) Work-related stress and how to manage it. https://www.hse.gov.uk/stress/causes.htm. Accessed 8/9/22.

61. Rajae-Joordens, R. and Engel, J. (2005) Paired comparisons in visual perception studies using small sample sizes. Displays, 26(1), 1-7. https://www.science direct.com/science/article/pii/SO14183820400708

For peer review only

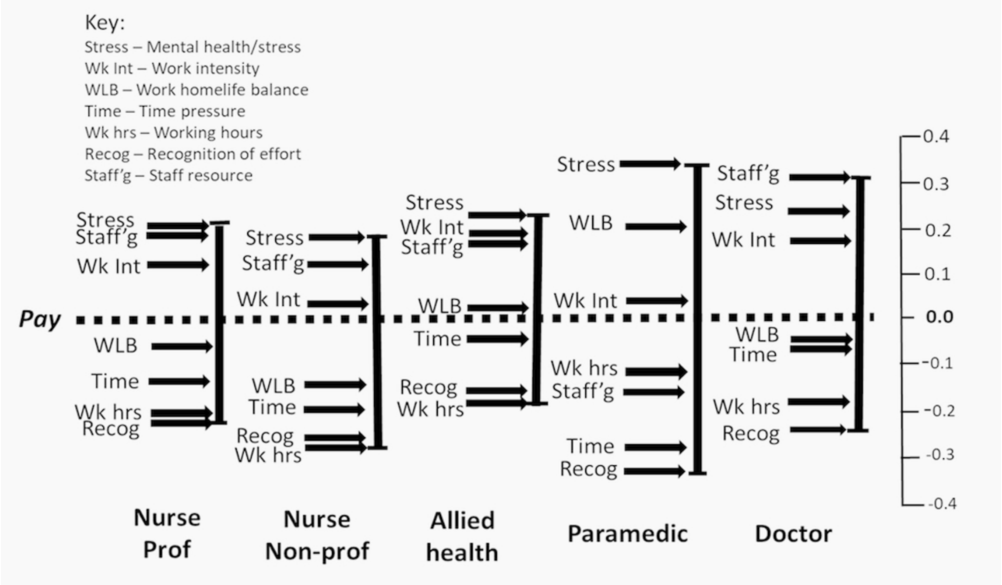

Figure 1 Weightings of push variables referenced to pay, by profession

707x413mm (72 x 72 DPI)

Appendix 1

Details of Push Variables ranking question

With refence to perceptions and beliefs relating to colleague behaviour, for a common set of variables (listed below).

Under the heading *'Why Do Staff Leave the NHS?' participants were presented with request::*

'We would like your views in more detail on how important the following issues are to explain why (RESPOPNDENT'S OCCUPATIONAL GROUP AUTOMATICALLY INSERTED HERE) staff leave the NHS. To make the task easier you will be presented with the issues two at a time.**'**

When each pair is presented, choose the issue that you think is the more important reason for (RESPOPNDENT'S OCCUPATIONAL GROUP AUTOMATICALLY INSERTED HERE) staff leaving the NHS.

Item set:

A. Time pressure
B. Staffing levels
C. Working hours
D. Workload (intensity of work)
E. Work/home-life balance
F. Pay
G. Mental health/stress
H. Recognition of contribution

Permutations of pairings (*N* = 28)

A&B; A&C; A&D; A&E; A&F; A&G; A&H

B&C; B&D; B&E; B&F; B&G; B&H

C&D; C&E; C&F; C&G; C&H

D&E; D&F; D&G; D&H

E&F; E&G; E&H

F&G; F&H

G&H

The data was gathered using an on-line platform hosted by YouGov. To mitigate presentation order bias, the order of pairings, as well as the order within each pairing (i.e. A&B or B&A) was randomised.

For peer review only

For peer review only

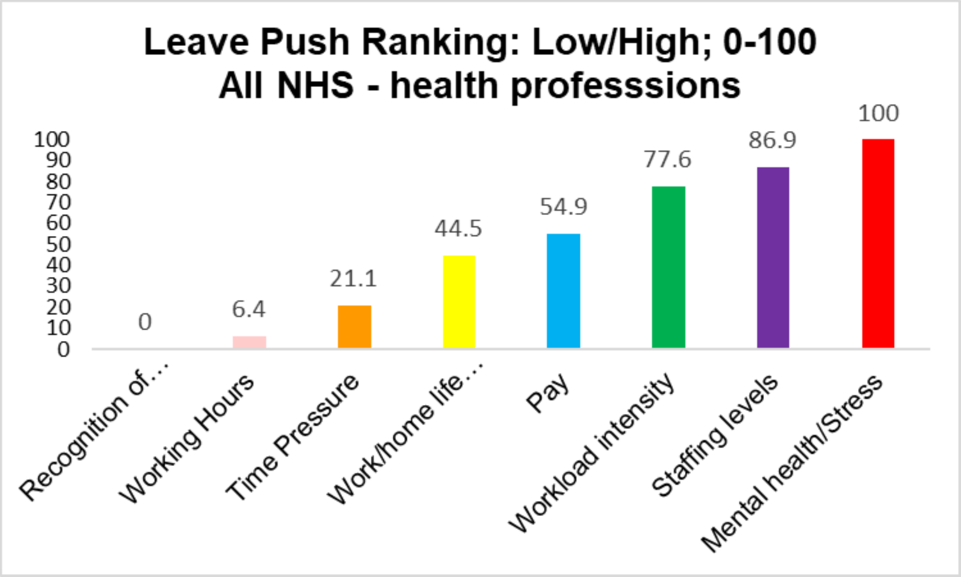

Relative salience of push variables – all NHS Health Professions

For peer review only

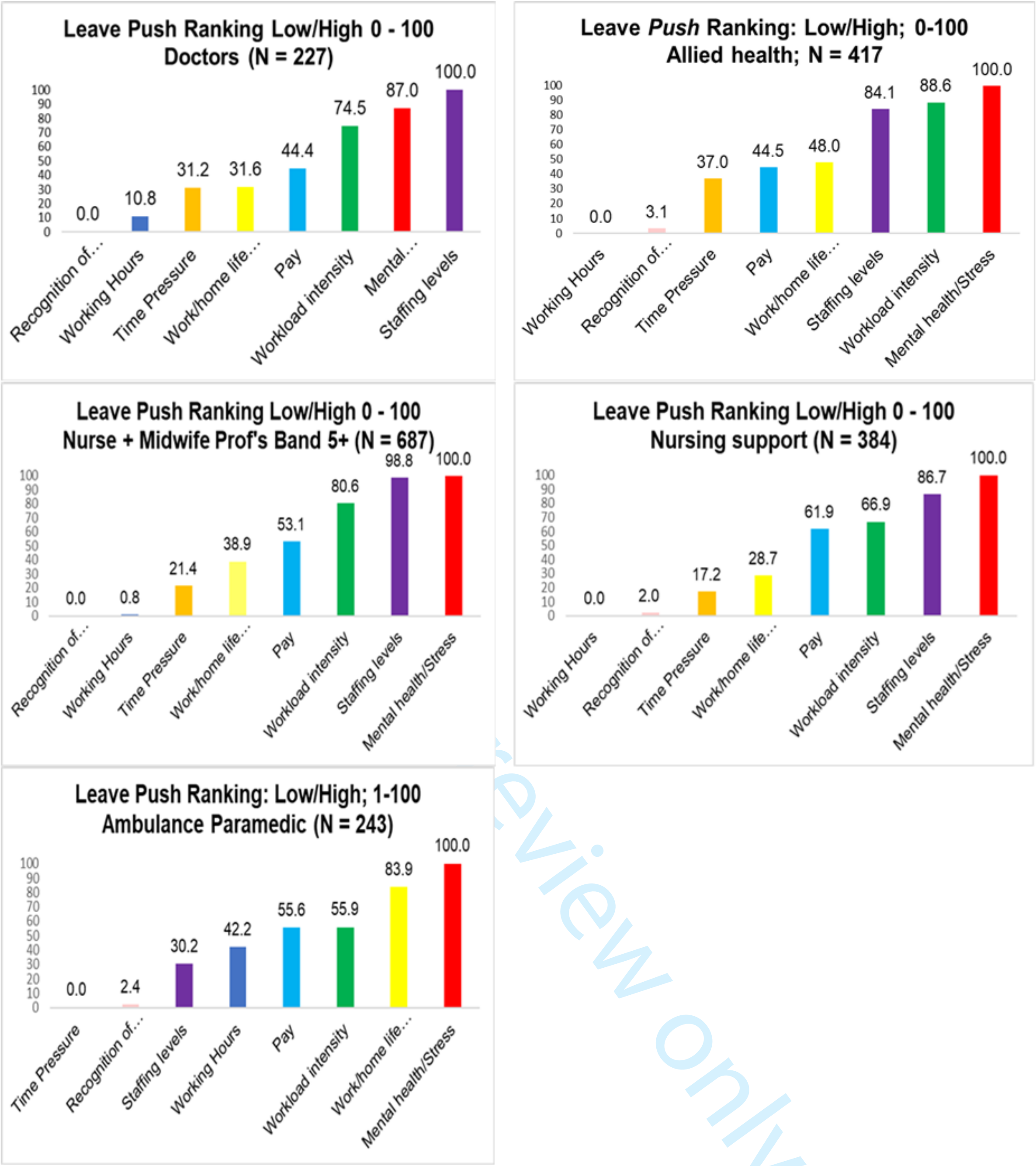

Relative salience of push variables – by Profession job-family

review only

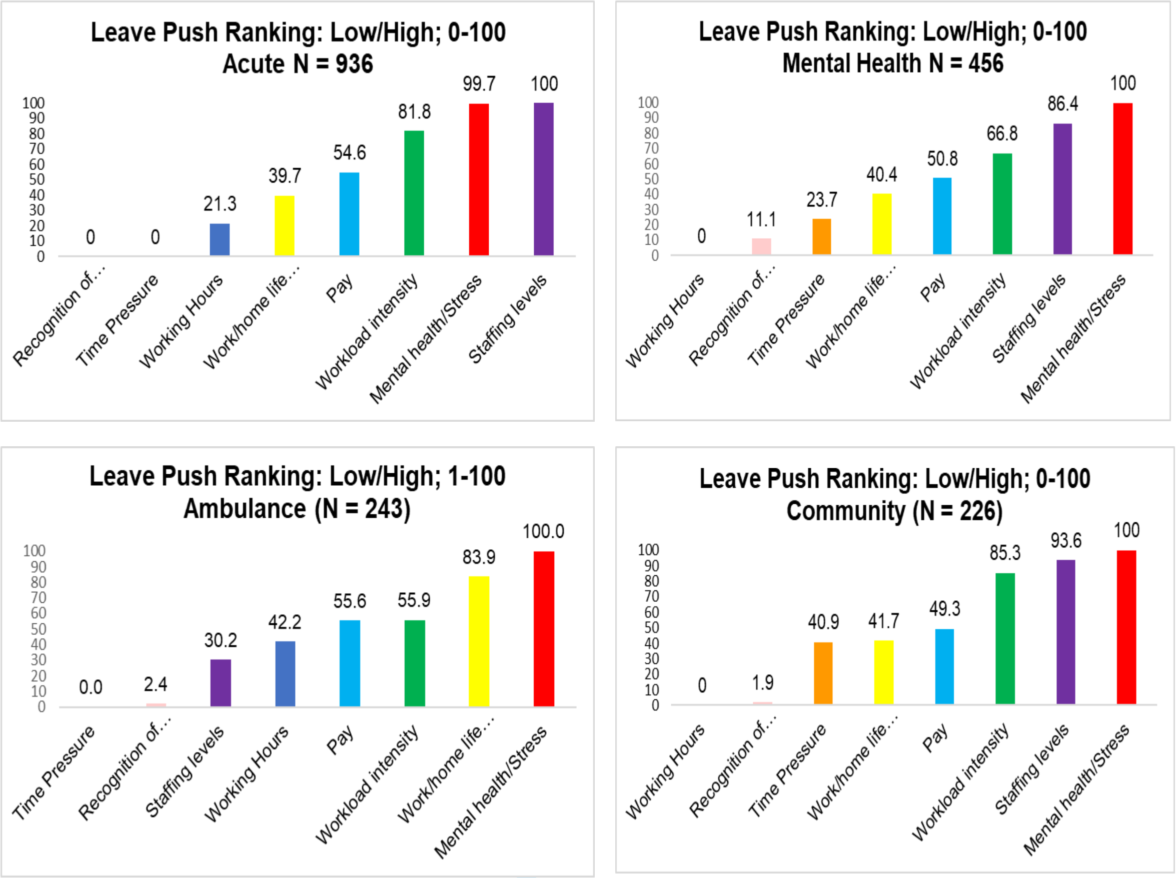

Relative salience of push variables – by care-provider organisation type

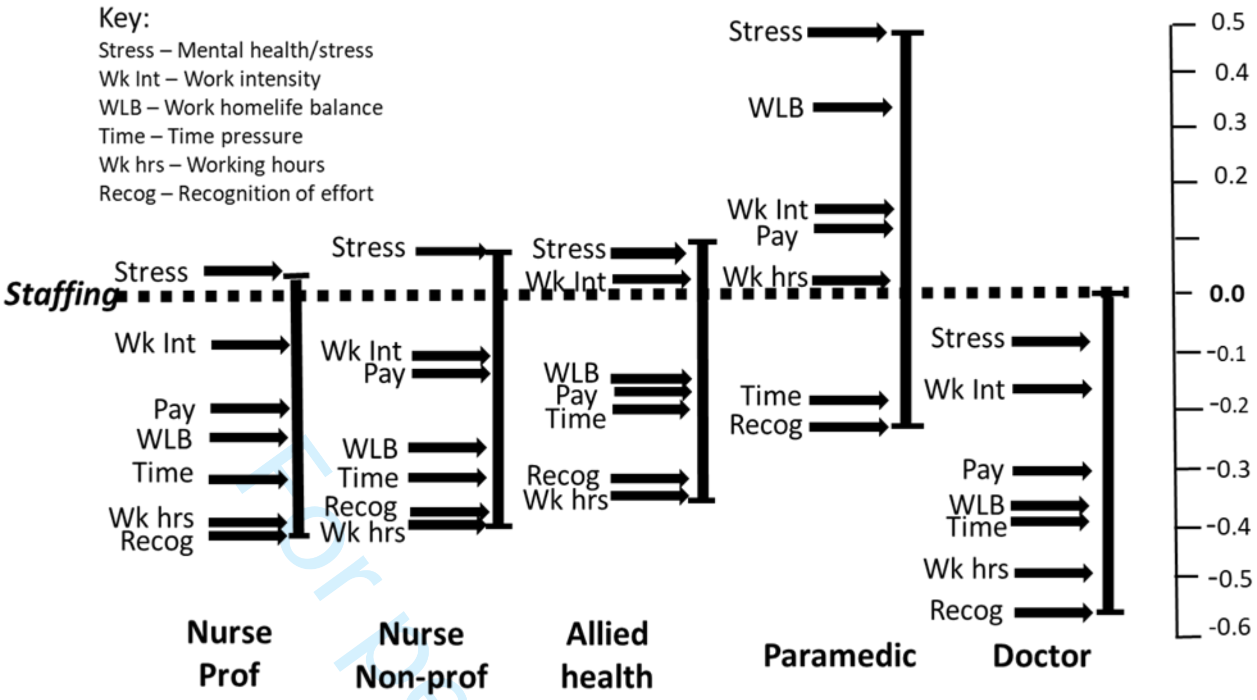

Weightings of *push* variables referenced to staffing resource level, by profession

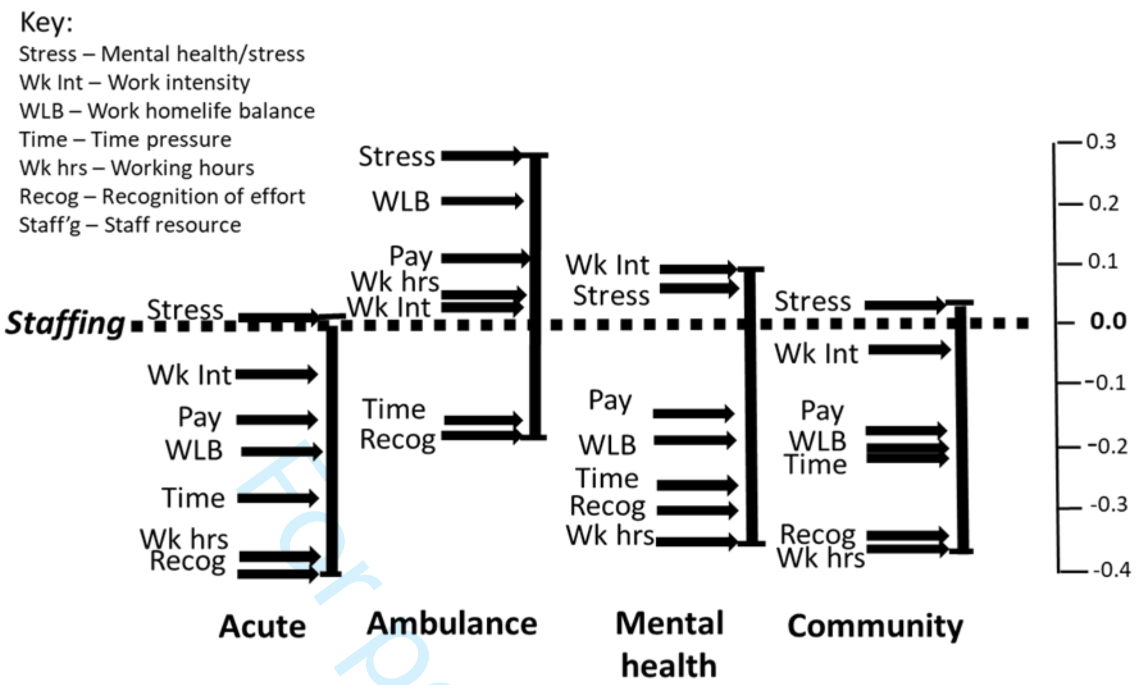

Weightings of *push* variables referenced to staffing resource level, by care provider organisation type

For peer review only

BMJ Open

Determining the relative salience of recognised push variables on Health Professional decisions to leave the UK National Health Service (NHS), using the method of paired-comparisons.

Journal:	*BMJ Open*
Manuscript ID	bmjopen-2022-070016.R2
Article Type:	Original research
Date Submitted by the Author:	05-Jul-2023

Complete List of Authors:	Weyman, Andrew; University of Bath, psychology<br>O'Hara, Rachel ; The University of Sheffield, School of Health and Related Research<br>Nolan, Peter; University of Leicester, School of Management<br>Glendinning, Richard; University of Bath, psychology<br>Roy, Deborah; University of Bath, psychology<br>Coster, Joanne; The University of Sheffield

**Primary Subject Heading**:	Health services research
Secondary Subject Heading:	Health policy
Keywords:	Change management < HEALTH SERVICES ADMINISTRATION & MANAGEMENT, Health & safety < HEALTH SERVICES ADMINISTRATION & MANAGEMENT, HEALTH SERVICES ADMINISTRATION & MANAGEMENT, Organisation of health services < HEALTH SERVICES ADMINISTRATION & MANAGEMENT, STATISTICS & RESEARCH METHODS, OCCUPATIONAL & INDUSTRIAL MEDICINE

SCHOLARONE™
Manuscripts

For peer review only - http://bmjopen.bmj.com/site/about/guidelines.xhtml

I, the Submitting Author has the right to grant and does grant on behalf of all authors of the Work (as defined in the below author licence), an exclusive licence and/or a non-exclusive licence for contributions from authors who are: i) UK Crown employees; ii) where BMJ has agreed a CC-BY licence shall apply, and/or iii) in accordance with the terms applicable for US Federal Government officers or employees acting as part of their official duties; on a worldwide, perpetual, irrevocable, royalty-free basis to BMJ Publishing Group Ltd ("BMJ") its licensees and where the relevant Journal is co-owned by BMJ to the co-owners of the Journal, to publish the Work in this journal and any other BMJ products and to exploit all rights, as set out in our licence.

The Submitting Author accepts and understands that any supply made under these terms is made by BMJ to the Submitting Author unless you are acting as an employee on behalf of your employer or a postgraduate student of an affiliated institution which is paying any applicable article publishing charge ("APC") for Open Access articles. Where the Submitting Author wishes to make the Work available on an Open Access basis (and intends to pay the relevant APC), the terms of reuse of such Open Access shall be governed by a Creative Commons licence – details of these licences and which Creative Commons licence will apply to this Work are set out in our licence referred to above.

Other than as permitted in any relevant BMJ Author's Self Archiving Policies, I confirm this Work has not been accepted for publication elsewhere, is not being considered for publication elsewhere and does not duplicate material already published. I confirm all authors consent to publication of this Work and authorise the granting of this licence.

For peer review only

Determining the relative salience of recognised *push* variables on Health Professional decisions to leave the UK National Health Service (NHS), using the method of paired-comparisons.

*Andrew Weyman, Reader, Department of Psychology, University of Bath, Bath, UK.

Rachel O'Hara, Senior Lecturer in Public Health, School of Health and Related Research (ScHARR), University of Sheffield, Sheffield, UK

Peter Nolan, Professor, School of Management, University of Leicester.

Richard Glendinning, Research Fellow, Department of Psychology, University of Bath.

Deborah Roy, Research Associate, Department of Psychology, University of Bath.

Joanne Coster, Research Fellow, School of Health and Related Research (ScHARR), University of Sheffield, Sheffield, UK

Corresponding author:
Dr Andrew Weyman
University of Bath
Department of Psychology
Claverton Down
Bath BA2 7AY UK
a.weyman@bath.ac.uk
Tel: 01225 385279

Word count: 4718

Keywords: Employee–retention, NHS, COVID-19, Intervention, Health professionals.

For peer review only

ABSTRACT

Objective: The primary and secondary impacts from the COVID-19 pandemic are claimed to have had a detrimental impact on health professional retention within the UK National Health Service (NHS). This study set out to identify priorities for intervention by scaling the relative importance of widely cited push (leave) influences.

Design: During Summer/Autumn 2021, a UK-wide opportunity sample (N=1958) of NHS health professionals completed an on-line paired-comparisons exercise to determine the relative salience of: work-related stress, workload intensity, time pressure, staffing levels, working hours, work-homelife balance, recognition of effort and pay as reasons why health professionals leave NHS employment.

Setting: The study is believed to be the first large-scale systematic assessment of factors driving staff exits from the NHS since the COVID-19 pandemic.

Results: All professions gave primacy to work-related stress, workload intensity and staffing levels. Pay was typically located around the mid-point of the respective scales; recognition of effort and working hours were ranked lowest. However, differences were apparent in the rank order and relative weighting of push variables between health professions and care delivery functions. Ambulance paramedics present as an outlier, notably with respect to staffing level (F-stat 4.47, *p*.004) and the primacy of work-homelife balance. Relative to staffing level, other push variables exert a stronger influence on paramedics than nurses or doctors (*f* 4.29, *p*.006).

Conclusion: Findings are relevant to future NHS health professional retention intervention strategy. Excepting paramedics/ambulance services, rankings of leave variables across the different health professional families and organisation types exhibit strong alignment, at the ordinal level. However, demographic differences in the weightings and rankings, ascribed to push factors by professional family and organisation type suggests that, in addition to signposting universal (all-staff) priorities for intervention, bespoke solutions for different professions and functions may be needed.

For peer review only

Article summary

Strengths and limitations of this study

- This is a large scale systematic assessment of the relative strength of widely cited *push* factors underpinning health professional exit from NHS employment since the manifestation of the COVID-19 pandemic.
- A key strength of paired comparisons, is that the output is an interval scale, that provides a quantifiable indication of the distance between the items, in this instance the relative strength of push influences.
- Differences in the rank order and relative weighting of *push* variables between the principal NHS health profession families' are identified.
- Although large for a variable ranking study of this type, the sample size was not sufficient to support multi-variate exploration of all permutations of potential interactions between the health profession demographics and push variables of interest.

For peer review only

BACKGROUND

NHS staff vacancy statistics for 2022 show an increase from approximately 133,100 full-time equivalent (FTE) staff in the quarter to June 2022 to around 133,400 in the quarter to September 2022. This represents a 5-year high (data are not available prior to the quarter from April – June 2017). The overall vacancy rate in the quarter to September 2022 stood at 9.7%, which also represents a 5-year high[1]. There are widespread claims, aligned with more substantiative evidence that the experience of working through the pandemic has significantly diminished health professional resolve/capacity to remain in NHS employment[2-4]. NHS staff survey data (2020)[5] shows a 44% increase in the proportion of staff reporting work-related stress, and around a fifth reporting considering exiting NHS employment, both of which have been attributed to the pandemic experience[6]. There are also reports of significant numbers of staff, notably junior doctors and nursing assistants leaving to take-up better paid jobs offering better working conditions outside the NHS[6,7]. It is important to note that the disparity between staff expressions of intention to quit and leaving behaviour can reflect systematic bias, potentially exaggerating the potential number of leavers. However, as a source of common error across successive waves of the NHS staff trend data can be considered more reliable.

The pent-up demand for care alone has created a major imbalance between demand for treatment and the capacity to deliver[8]. Recognition of the finite scope for recruitment of migrant labour, and the inevitable time-lag in training new health professionals, combined with institutional worry over the likely magnitude of pandemic associated exits has given rise to an unprecedented focus NHS policy makers on finding ways to stabilise/enhance staff retention. This paper reports

For peer review only

findings from a study of the relative salience and weighting of an array of widely cited *push* influences on health professional exit from NHS employment.

From the perspective of intervention aimed at enhancing retention rates, it is important to know which variables exert the strongest push, and their relative weightings. However, it is also important to determine whether this profile varies across different segments of the workforce i.e. whether 'one-size-fits all' or bespoke interventions are needed[9]. Almost all previous studies of NHS staff retention are unable to answer this question, as their samples are typically be limited to single professions or institutions[10].

The study is the first large scale systematic analysis of the precursors to exit following the emergence of COVID-19. The findings are relevant to human resource intervention strategy aimed at stabilising and enhancing staff retention. They provide evidence of which variables constitute the greatest influence on employee leave decisions and the degree to which they present as universal (all staff) or variable across different health profession and organisation type demographics. The focus here is on health professional migrations to non-NHS employment, as distinct from transitions from one NHS employer to another, the former representing a loss to State sector capacity, the latter producing no net change. This key distinction is frequently overlooked or blurred in reports of NHS staff turnover. The research builds on the research team's previous work within the sector[10] and broader published findings on reasons why health professional leave NHS employment.

Established insights

For peer review only

Pre-pandemic systems perspective research focused on the role of extrinsic aspects of work in eroding job satisfaction, mental health, physical health and strength of attachment to the NHS, notably job-demands, workload, pay, resources, and configuration of working hours[11-13]. The perspective, in essence, is a risk-based attrition model focused on the detection of precursor relationships. A high proportion of study findings point to shortage of resources, particularly staff resources as a, if not *the,* cornerstone element harbouring the potential to produce an array of detrimental ripple effects. Staff shortages tend to produce time pressure, increased workload, and work rate, norming of (paid and unpaid) overtime working, with implications for employee fatigue, work-homelife balance and sickness-absence rates, mental health and burnout[14-17]. Related psychosocial effects identified include corrosive effects on morale, frustration and concern over compromised standards of patient care[18,19], amplified worry over capacity to cope, making errors and, relatedly, anxiety over personal professional vulnerability[20-23].

At a fundamental level, Issues of high/excessive workload can be conceptualised as reflecting an imbalance between the demand for patient care relative to resource, although other climate and culture factors can play a role, e.g. claims of increased performance monitoring, other forms of auditing and accountability related bureaucracy, have also been reported as notable sources of health professional frustration and disaffection[24,25]. Contributions from systems perspectives on mental health and work-related stress are also extensive, and principally emphasise the role of contextual effects arising from the design and configuration of work, i.e. extrinsic elements of job-demands, that embody the potential to cause psychological harms that erode staff disposition/capacity to remain[26,27].

For peer review only

Findings on the role of pay in exit decisions are mixed. Some claim it as a primary criterion[28,29], whereas others report it as less important than workplace climate variables, notably sufficiency of resources and staffing levels[30,31,13]. Pay is relevant in both absolute and relative terms, with linkages to notions of fairness, equity and recognition of effort[32-35]. As Dean, 2011[36] notes, dissatisfaction with pay tends to be amplified where rates remain static and are perceived by employees to be disproportionate to effort expended, in the presence of significant rises in work rate and workload; this being the case since the emergence of COVID-19.

The issue of working hours, with respect to shift duration, flexibility over their configuration and the availability of part-time work has received extensive attention[37]. Findings relating to the widespread adoption of compressed hours and associated increases in typical shift duration (typically 10-12 hours) over recent decades within the NHS are mixed, liked by some staff while disliked by others[38,39,40], Irrespective of employee preferences, there are claims of long-term detrimental impacts on health, and amplified fatigue amongst older workers[37]. Increasing the availability of flexible and part-time hours is widely cited as a means of enhancing retention[39,41,42, 43].

The analysis that follows measures NHS health professionals' ratings of the relative salience of headline push influences on exit decisions after 12 months of exposure to COVID-19 working conditions.

For peer review only

The aim of the study was to provide future human resource policy-relevant insight into issues and priorities for intervention to sustain/enhance NHS staff retention rates.

The operational objectives were: (i) To produce on ordinal high-low ranking of headline *push* variables. (ii) To produce scaled output of the relative weighting of headline push variables. (iii) To determine the degree to which (i) and (ii) vary by health profession family, and type of secondary care organisation (acute; mental health, community and ambulance).

METHOD

Patient and public involvement

None. There was no patient or public involvement in this study as its focus was on NHS employees, only. Participants in the study have access to a report on headline findings from the UK-wide survey of NHS staff. The authors would like to thank members of the research stakeholder advisory group involving representatives from NHS England/Improvement, health sector professional associations and trades unions for their guidance and feedback on the content of the UK-wide survey of NHS staff of which the paired ranking exercise was a component.

Data collection

The data was gathered from July to December 2021, as a component of the second wave of the authors UK-wide on-line quantitative survey of influences on NHS staff

For peer review only

retention[44,45]. Ethical approval for the research was granted by University of Bath Department of Psychology Ethics Committee (reference number: PREC 20-259); and [NHS ethics] the research received National Institute for Health research (NIHR) portfolio adoption. Respondent participation was voluntary and anonymous, and all participants provided informed consent prior to taking part.

Mirroring previous research into NHS staff retention by the authors[10] the method selected for the study was paired-comparisons[46]. Paired-comparisons is well suited to eliciting views on multi-faceted subjective variables. The cognitive load on participants is low (each is requested to select one item from a randomly presented pair of items, and to repeat this for all permutations of pairings within the set). Its key strength relative to alternatives, e.g. direct ranking, subjective rating scales and sorting techniques such as Q.Sort and repertory grid, is that the output is an interval scale, that provides a quantifiable indication of the distance between the items[46,47,48], in this instance the relative salience of *push* influences.

(i) Generation of the item set

A set of eight widely referenced *push* variables were selected[16,49,50], six of which were common to a previous study of NHS staff retention[10]: *staffing levels*, *working hours*, *mental health/stress*, *pay*, *time pressure*, and *recognition of contribution*. *Workload intensity* and *work-life balance* were added to the item set following consultation with government, employer, professional body and trades union stakeholders, and to take account of the prevailing post-pandemic working conditions, *workload intensity* and *work-life balance* were added to the item set.

For peer review only

(ii) Reference criterion

The study set out to tap health professionals' insight into and perceptions of the relative importance of a set of widely cited reasons why colleagues within their health profession / job role leave NHS employment. Specifically, participants were asked *'how important the following issues are to explain why* (their health profession inserted) *staff leave the NHS'.*

This produced a set of subjective scales of push effects. The purpose was not to determine rates of exit, but to compare how staff beliefs about these push factors might vary between different health professional families and types of care provider organisation. The underpinning rational here was twofold. First, asking staff about their own future leave versus stay intentions was not considered a robust option as it tends to exaggerate leaving rates to an unknowable degree, due to recognised intention behaviour disparities [51], i.e. people often don't achieve their intentions. Asking participants about the behaviour of colleagues cannot be considered free from bias or error, as it is subjective, but the error can be considered common across the different groups of personnel. . Secondly, from the perspective of identifying human resource priorities for intervention aimed at stabilising/improving retention rates it is important to know the relative strength of the push factors in the item set as these can be considered to operate as precursors to exit.

(iii) Participants

Participants were a UK-wide volunteer sample recruited via the YouGov panel; UNISON trade union members and 12 NHS Trusts in England using electronic

For peer review only

mailing and newsletter communications to distribute a link to the online survey. A breakdown of the sample by health profession job-family is provided in table 1.

Table 1 Breakdown of sample by job- family

**Profession job-family**	**Numbers Employed (NHS England only)**	**Paired ranking sample**
Doctors (consultant & specialist inc trainees)[1]	124,000 (15%)	227 (12%)
Nurse professionals & Midwives[2]	332,000 (40%)	687 (35%)
Nurse non-professional[3]	280,000 (33%)	384 (20%)
Allied Health[4]	84,000 (10%)	417 (21%)
Ambulance[5]	18,000 (2%)	243 (12%)
**Total**	**837,000**	**1958**

Source: 1,2,4,5 NHS England Staff in post statistics June 2022 https://app.powerbi.com/view?r=eyJrIjoiYTlhMTAzNDEtMzQ2Ny00MDRjLWIyZTAtMTY2ZGVlZmJhMjEwIiwidCI6IjUwZjYwNzFmLWJiZmUtNDAxYS04ODAzLTY3Mzc0OGU2MjllMiIsImMiOjh9
4 Michas, F. (2022) Statista https://www.statista.com/statistics/679783/number-of-allied-health-professionals-in-nhs-workforce-england/

(iv) Procedure

Participants were presented with pairs of push factors, for all permutations of the eight factors; 28 for each participant. The order of presentation of pairs was randomised. For each pair, participants were asked *'Which of these two factors is the bigger influence on why staff in your profession / job role' leave the NHS'* (see appendix 1).

(v) Pre-analysis check of within respondent consistency

To consider the data set suitable for scale development, it is important to determine that participants are able to make consistent judgements, i.e. evidence that the stimulus items produced logically inconsistent output, of the type A>B>C>A, would suggest that the item set does not scale, and would be unsuitable for scale development. Therefore, tests of within-respondent consistency (Kendall's K) were performed on a pilot sample of response sets (n = 60). This revealed that 94% of

For peer review only

response sets exhibited high consistency (*K* =/>0.70). The item set therefore was judged to be suitable for reliable scale development[46,47].

ANALYSIS

The analysis took the form of an iterative complementary approach.

(i) Generation of an NHS-wide all-profession family interval scale to demine the global profile of relative salience of the push variables in the item set (see appendix 1).

(ii) Generation of separate, dedicated, health profession family and care delivery organisation type scales, to determine the degree of homogeneity/heterogeneity in the rank orders and weightings of the set of push variables between these demographics.

(iii) Generation of push variables scales relative to pay and staffing levels, by health profession family and care delivery organisation type.

(iv) Formal (statistical) testing of the degree of variability in the collective strength of push variables relative to pay and staffing level across different demographics, i.e. do variables within the domain of reasons to leave constitute a stronger push with some professions/organisation types than others.

Global (all NHS health professions) ranking of push variables

In the first instance, a global all-NHS (all profession/all delivery function) interval scale of the relative importance of *push* influences was produced. Judgement proportions were determined and means for each *push* were calculated. Setting the

For peer review only

lowest ranked variable (*recognition of contribution*) to zero, and the highest (*mental health/stress*) to 100 produced the relative weightings depicted in appendix 2.

Findings point to the primacy of *mental health/stress* and *staffing levels*, *pay* is ranked fourth of the eight push variables and *recognition of contribution* and *working hours occupy* the two lowest ranks. When interpreting the findings, it is important to keep in mind that they relate to a scaling of widely cited headline drivers of exit, i.e. a scaling of *important push* influences. As such, a low ranking is not synonymous with being unimportant.

Having established the global profile, deeper analyses explored differences by health profession and type of care provider organisation.

Health profession (job-family) contrasts

Contrasts were explored between doctors (consultant & specialists, including trainees), nurse professionals and midwives, nurse non-professional, allied health professionals, and ambulance paramedics (including technicians); see table 1 (scientific and technical professionals were excluded due to restricted sample size) and four types of care provision setting: acute, mental health, community and ambulance services.

This analysis revealed notable alignment between profession groupings but also difference in rank orders and respective weightings. Mirroring the global profile, mental health/stress; staffing levels and workload intensity dominated the top three ranks across the different professions. However, staffing level was ascribed a notably less prominent position by ambulance participants (see appendix 3). Formal

For peer review only

testing (ANOVA) revealed that that contrast with the other job families was statistically significant (F-stat 4.74, p-value .004).

Working hours, recognition of effort and time pressure consistently occupied the three lowest ranks across all profession families, except ambulance services where working hours was ranked fifth and ascribed a markedly higher weighting. Pay consistently occupied the mid-range, being ranked 4th or 5th across each of the occupation groupings, although having a higher weighting amongst nursing (most notably amongst nursing support grades; job bands 1-4) and ambulance service paramedics. The most marked contrast between the profession families related to the relative weighting of work-home life balance. Again, ambulance service paramedics present as an outlier, ranking this the second strongest influence on exit with a notably higher weighting (83). There was high consistency with respect to the ranking (4th or 5th) of this variable across the other professions, although allied health gave it a higher weighting (48) than doctors (31.6), nurse/midwife professionals (35.5) and nursing support (28.7).

Correlation (Pearson *r*) and linear regression (R^2) analyses, comparing variable ratings for all permutations of pairings of job-family, show high alignment across all groups (*r* .93 -.99; R^2 .88 -.97) with the exception of paramedics which, present as having a different profile (*r* .57-.72; R^2.44 -.53). An alternative, and complementary means of comparing the degree of between group agreement is to compare the degree of alignment at the ordinal level. Formal testing of this (kappa coefficient *K*) highlighted high congruence between nursing staff (professional and non-professional) and doctors (*K*= .75), but modest to low alignment between other groups (Nurse professional v Allied health; Doctors v Nurse non-professional, *K*= .50; Nurse professional v Allied Health v Doctors, K=.375 all other contrast *K*= .25).

For peer review only

Confirming the regression analysis, the most marked contrast was found between paramedics and each of the other occupations.

Service provider organisation contrasts

The findings indicated high alignment by type of care provider organisation at the level of rank order, excepting ambulance services, which again present as an outlier. However, a number of contrasts are apparent with respect to the weighting, notably *work intensity* in acute hospitals and community; *time pressure* in community and *recognition of effort* in mental health settings (see appendix 4).

Correlation analyses of variable ratings for all permutations of pairings of care provider organisation show a contrast between ambulance service and the other functions (Acute v Mental health; Acute v Community; Mental health v Community r=>0,9, R^2>0.90). Ambulance services v's Acute, Community and Mental health, r=0.50 -.62; R^2 .30 -.38). The other three functions show strong alignment over the five highest ranked variables, with Mental health and Community sharing a common order of priority.

Weightings of *push* variables relative to staffing resource and pay

Reflecting Thurstone's five method[46], the analysis proceeded to scaling the push variables relative to knowable objective value anchors of staffing levels and pay. For these separate analyses, the respective anchor item (pay or staffing) was set to zero. This produced a push scale for each profession and type of care provider organisation. Formal testing of between group differences (ANOVA) afforded insight

For peer review only

into whether the set of headline push influences relative to pay (and staffing level) exert stronger collective leverage on some professions and care provider organisation types than others.

Staffing levels

Table 2 gives the relative weightings by job-family. It highlights paramedics/ ambulance services as an outlier. The profession contrasts revealed a statistically significant difference between paramedics and doctors and paramedics and qualified nurses (F-stat 4.29), (p-value .006) but did not exceed the .05 threshold in comparison to allied health and nursing support).

Table 2. Normalised assessment values calculated from paired-comparisons made by five NHS health profession families

Push variable	Nurse Prof Job band 5+	Nurse non-prof Job band 4-	Allied health	Paramedics	Doctors
Time pressure	-0.331	-0.328	-0.197	-0.193	-0.402
Working Hrs	-0.421	-0.413	-0.356	0.0004	-0.521
Work intensity	-0.081	-0.096	0.020	0.143	-0.159
Work-home balance	-0.257	-0.274	-0.151	0.330	-0.400
Pay	-0.197	-0.120	-0.166	0.117	-0.328
Mental health	0.0055	0.069	0.070	0.485	-0.084
Recognition	-0.425	-0.402	-0.342	-0.253	-0.589
Staffing level	0.000	0.000	0.000	0.000	0.000

Graphical representations of these findings depicting the relative distance between push variables are provided in appendix 5.

For peer review only

Contrasts by type of care provider (table 3) showed a significant difference between ambulance and the other care provider functions, Acute (F-stat 3.98), (p-value .018); Community (p-value .040) and Mental health (p-value .056).

Table 3. Normalised assessment values calculated from paired-comparisons made by four NHS health care provider organisation types

Push variable	Mental health	Acute	Community	Ambulance
Time pressure	-0.283	-0.323	-0.228	-0.193
Working Hrs	-0.396	-0.412	-0.411	0.0004
Work intensity	-0.090	-0.078	-0.037	0.143
Work-home balance	-0.208	-0.248	-0.225	0.330
Pay	-0.162	-0.188	-0.193	0.117
Mental health	0.066	-0.001	0.029	0.485
Recognition	-0.342	-0.413	-0.402	-0.253
Staffing level	0.000	0.000	0.000	0.000

Graphical representations of these findings depicting the relative distance between push variables are provided in appendix 6.

Pay

In the case of pay, contrasts were explored in relation to profession and (where the sample size was sufficient) by grade, in the case of nurses (professionals, versus nursing support). Table 4 gives the relative weightings by job-family.

For peer review only

Table 4. Normalised assessment values calculated from paired-comparisons made by five NHS health profession families

Push variable	Nurse Prof Job band 5+	Nurse non-prof Job band 4-	Allied health	Paramedics	Doctors
Time pressure	-0.134	-0.208	-0.031	-0.309	-0.074
Staffing level	0.197	0.119	0.166	0.116	0.328
Working Hrs	-0,244	-0.292	-0.191	-0.116	-0.193
Work intensity	0.116	0.024	0.185	0.026	0.169
Work-home balance	-0.060	-0.154	0.014	0.213	-0.071
Mental health	0.203	0.189	0.235	0.369	0.244
Recognition	-0.228	-0.283	-0.176	-0.369	-0.261
Pay	0.000	0.000	0.000	0.000	0.000

Formal testing (ANOVA) revealed no statistically significant differences (*F*-stat 0.35, p-value .842). A graphical representation of the relative distance between pay and the other push variables by job-family is provided in figure 1.

[Insert figure 1 about here]

Figure 1 Weightings of *push* variables referenced to pay, by profession

DISCUSSION

Determining the relative strength of headline factors driving early-exit offers the promise of informing strategic decision-making respect to ameliorative intervention both by government and NHS employers. Insights in this area are of central relevance to signposting which push effects constitute priority issues for intervention with the potential to deliver the highest impact on health professional retention and future NHS capacity. The overarching finding is that mental health/stress; staff resources and workload show primacy in the scaling of push influences. Pay and

For peer review only

work-homelife balance occur within the mid-range of the distribution, and recognition of effort, and working hours occupy the lowest positions.

Beyond almost universal consensus over the primacy of mental health/stress, and the basal position ascribed to working hours and recognition of effort, a number of health profession and organisation type demographic contrasts are apparent in the rankings and relative weightings ascribed to push influences. The most marked contrast is between the paramedic/ambulance services profile and other occupations/functions. Staffing was ranked sixth by paramedics, compared with first or second and work-homelife balance was ranked second, compared with fourth or fifth by each of the other occupations. The weighting ascribed to staffing by the other professions indicates that it exerts a stronger push on doctors, allied health, and nurse professionals than non-professional nurses, and paramedics. The higher salience of work-life balance to paramedics may reflect differences in the (ir)regularity and duration of their working hours. Emergency ambulance crews have markedly more irregular shift patterns, combined with routinised over-time working. A significant proportion of their overtime is attributable to shift 'over-run's', i.e. ad-hoc, unplanned and involuntary extensions to the working day/incursions into non-work time[52]. The higher weighting of working hours by paramedics compared with the other professions aligns with this conclusion.

The much lower rank ascribed to staffing resource by paramedics is perhaps the most noteworthy finding, in view of its primacy in the scales produced by each of the other personnel groups/functions, as well as the strength of objective evidence of significant shortage of paramedics[53]. It is possible that the relative social isolation of

For peer review only

paramedics has the effect of making the issue of staff shortages less cognitively-available, i.e. less visible and immediately apparent in their work environment, than would be the case in a hospital context. Accepting this, a similar profile might have been expected from community care respondents, a high proportion of whom are also subject to peripatetic lone working, but this was not apparent. On balance, it seems questionable that social isolation would be sufficient to significantly attenuate employee awareness of staff shortages, not least because of its visible secondary impacts, i.e. increased pressure to work overtime, threats to the realisation of Trust call-out targets, as well as regular high profile coverage of staff shortages within ambulance sector publications and mass media[54,55,56].

Relative to staffing, stress, work-home life balance, work-intensity, pay and working hours exert a stronger push influence amongst paramedics than the other professions, i.e. within the leave domain these variables exert a relatively stronger push for paramedics than other professions. This explanation rests upon the premise that staff shortages can be treated as constant across each of the professions and care delivery functions, a shared benchmark against which other push variables can be scaled. If this interpretation is accepted, it suggests that attention to paramedic retention represents a key priority and source of vulnerability to the NHS. The finding from the research team's broader survey of NHS staff retention that 24% of paramedics (N= 1157)[44] reported having submitted one or more applications for a non-NHS job in the previous six months, compared with the all-staff rate of 13% (N = 9220)[45] lends weight to this conclusion. The data does not permit a precise explanation for the marked difference in the paramedic response profile. However,

For peer review only

the evidence that the profile of precursors to exit for paramedics is different from the other professions and functions presents as strong.

In contrast to some contemporary media and industrial relations accounts[55,56,57], and some academic research findings[58], pay, as a reason to leave did not feature prominently. Ranked fourth/fifth by each of the professions, this profile mirrored finding from previous research on NHS staff retention[10], which concluded that dissatisfaction with pay as an incentive to leave needs to be considered with reference to opportunities for more remunerative employment elsewhere. UK Labour Force Survey data indicates that (excepting doctors and dentists and care assistants) pay rates achieved by NHS leavers are not significantly higher, and for certain professions (notably, paramedics), they are commonly lower[37,53,6]. While other variables appear to exert a stronger push than pay, this is not grounds to diminish it as a potential source of dissatisfaction in absolute terms. However, it does suggest that attention to pay alone is unlikely to fix the retention issue.

The finding that working hours was ascribed a low rank and weighting across all professional groups and functions (again excepting paramedics) mirrors its profile in previous work on staff retention[16] and questions the emphasis it receives within contemporary policy and employer guidance publications[42,43]. Widely cited as attractive to all employees, but particularly millennials and staff with caring responsibilities, increasing the availability of part-time and flexible hours is cast as key to increasing retention rates. However, the findings suggest that its salience to leave decisions is lower than mental health, staff resource (excepting paramedics), work intensity, pay, and work-homelife balance. Mirroring the conclusions on pay,

For peer review only

this does not represent grounds to diminish the value of flexible work hours and its attractiveness to staff, but it does suggest that attention to this issue may have a modest impact in the absence of attention to more highly ranked and variables.

The findings point to the primacy of mental health/stress as the reason why staff leave. Mental health and stress are lag effects, that can be attributable to work and non-work variables, as well as intersects between the two, mediated by individual resilience. For employers, a comprehensive perspective on intervention means addressing root causes attributable to the configuration of work, as well as supporting individuals at times of need[59,60]. In contrast to stress, sufficiency of staff resources is an important lead (as well as a lag) indicator.. Sufficiency of resources has material impacts on job-demands, work-life balance, time pressure and working hours as well as psychosocial impacts on psychological stress and morale.

Strength and limitations

This study is believed to be the first large scale systematic assessment of *push* variables following manifestation of the COVID-19 pandemic and its secondary impacts. The findings provide insight into the relative weighting of headline reasons why health professionals leave NHS employment. The output is relevant to determining human resource priorities for intervention to stabilise and enhance staff retention rates.

The method of paired comparisons' produces an interval scale of the relative distance, within psychophysical space, between a set of related entities, which can also be anchored to respondent's subjective estimates of knowable quantifiable entities, e.g. pay or staffing level. Respondents were asked about their beliefs

For peer review only

regarding the exit behaviour of peers rather than their own intentions. Neither feature can be considered free from some degree unquantifiable error. However, each can be regarded as a source of common error, i.e. that *cancels-out,* when comparing the output profiles across different segments of the NHS workforce, in relative terms.

The findings reported within this paper are based upon an opportunity sample of voluntary participants with notable variability in the sizes of the occupation and care organisation type sub-samples. In common with the NHS annual staff survey, all other voluntary participation employee surveys, the potential for self-selection response bias cannot be discounted.

Proportionately, the profession job-family sub-samples show alignment with NHS contemporary staff-in post ratios for doctors, qualified nurses and midwives, but under-represent nurse non-professionals and over-represent allied health professionals and ambulance staff. However, the smallest sub-sample exceeded accepted norms regarding the minimum number of participants for scale development[61] by a factor of 10. Also, it is important to note than the scales of push factors are not subject to variability due to the sample size or influenced by variation between the proportions in realised sample and actual proportions within the NHS workforce.

Although large for a variable ranking study of this type, the sample was not sufficient to support multi-variate exploration of possible interactions between the health profession and organisation type demographics of interest.

For peer review only

CONCLUSIONS

Excepting paramedics, rankings of leave variables across the different health professional families exhibit a high degree of alignment, at the ordinal level, and highlight the primacy of psychological stress, staff shortages and work intensity. However, the presence of demographic differences in order of priority and weightings of push variables suggests that, in addition to signposting universal priorities for intervention aimed at stabilising and stemming prevailing exit rates, it is important to consider bespoke priorities/solutions for different professions and functions. Paramedics present as an outlier, exhibiting a profile that is significantly different to other health professionals. There may also be grounds for inferring that the leave variables explored exert a relatively stronger push on members of this group.

While increases in pay are transparently important to NHS staff, findings from this research suggest that enhancements in that domain alone may produce a modest impact on retention. An equivalent conclusion might be drawn with respect to the current high-profile emphasis on increased access to flexible working hours as a solution within contemporary NHS staff retention guidance to employers. Both have potential to *do-good*, but there are grounds for inferring there is a risk that neither may deliver sufficient *good* to redress the high and rising exodus in the absence of attention to what present as more fundamental factors driving exit. Importantly, scope for addressing the highest ranked factors driving exit, in large degree, lies beyond the gift of NHS employers.

For peer review only

The findings of this paper relate to the scaling of widely cited headline factors driving exit, i.e. a scaling of important *push* influences. As such, it would be unwise to interpret low ranked variables as unimportant. A more prudent interpretation would be to regard intervention to address low ranked variables as embodying potential to contribute to a comprehensive, multi-faceted programme of activity, but unlikely to produce the necessary degree of leverage in the absence of attention to more highly ranked, more fundamental, influences of staff exit behaviour.

For peer review only

RECOMMENDATIONS

- The insights from this study can contribute to the evidence base-for prioritising push issues and demographics for intervention aimed at stabilising / enhancing NHS health professional retention.
- NHS workface policy and planning functions could review the extent to which current staff retention strategies align with factors identified in this study.
- Further research / published evidence synthesis is needed to map the scope for intervention to address the higher ranked push factors.
- The set of push factors can be considered to constitute precursors to exit. NHS employers would benefit from a tool to benchmark and regularly (e,g, annually) monitor these variables in order to gain feedback on the effectiveness of their interventions and practices aimed at stabilising/enhancing health professional retention rates.

Funding statement
Economic and Social Research Council, No: ES/V015389/1.

Authors' contributions

AW, PN, RO and RG conceived and designed the study. AW led the data analysis and drafted the paper. DR and JC managed the process of securing necessary ethics and governance permissions to conduct the study. DR contributed to data analysis. All authors read drafts of the manuscript and approved the final version. AW is guarantor for the paper.

Acknowledgements

The authors would like to thank the interview participants. We are also grateful for the input of the project advisory group for their guidance and assisting with facilitating access to participants.

Competing interests

None declared - there are no competing interests for any author.

Data sharing statement

The data set can be made available to researchers

Ethics Approval

This study was approved the University of Bath, Department of Psychology Ethics Committee Reference number: 21-027

References

1. Charlesworth, A. (2022) NHS Vacancy Rates point to deepening workforce crisis The Health Foundation. https://www.health.org.uk/news-and-comment/news/nhs-vacancy-rates-point-to-deepening-workforce-crisis Retrieved 20/4/23

2. O'Dowd, A. (2021) *NHS staff's stress levels rose last year as covid pandemic took its* toll. BMJ 2021*;* 372: n703 https://doi.org/10.1136/bmj.n703 (12 March) Retrieved 4/9/22.

3. De Kock, J.H., Latham, H.A., Cowden, R,G., Cullen, B., Narzisi, K., Muñoz, S-A., Leslie, S.J., McNamara, N. and Boggon, A. The mental health of NHS staff during the COVID-19 pandemic: two-wave Scottish cohort study. Cambridge University Press: 07 January 2022. https://www.cambridge.org/core/journals/bjpsych-open/article/mental-health-of-nhs-staff-during-the-covid19-pandemic-twowave-scottish-cohort-study/F77D24E3A13A7C8BC89D16E3ABF637B1.

4. Oliver, D. Act on workforce gaps, or the NHS will never recover. BMJ 2022*;* 376: n3139*.* https://doi.org/10.1136/bmj.n3139 (05 January 2022)*.*

5. National Health Service. NHS Staff Survey 2021 staff survey data; March, 2022. https://nhsstaffsurveyresults.com/static/1f3ea5c952df62a98b90afcf3daa29ac/ST21-National-briefing.pdf Retrieved 12/10/22.

For peer review only

6. Anderson M, O'Neill C, Clark JM, Street A, Woods M, Johnston-Webber C, Charlesworth A, Whyte M, Foster M, Majeed A, Pitchforth E. Securing a sustainable and fit-for-purpose UK health and care workforce. The Lancet. 2021 May 22;397(10288):1992-2011

7. Wilson, H.C.P., Abrams, S., Simpkin-Begin, A. Drexit: Understanding why junior doctors leave their training programs to train overseas: An observational study of UK physicians. Health Science Reports 2021. Wiley on-line library. https://doi.org/10.1002/hsr2.419

8. Martin, G., Clarke, J. Markar, S., Carter, A.W., Mason, S., Purkayastha, S., Darzi, A., Kinross, J. How should hospitals manage the backlog of patients awaiting surgery following the COVID-19 pandemic? A demand modelling simulation case study for carotid endarterectomy 2020; https://doi.org/10.1101/2020.04.29.20085183

9. Karanika-Murray, M. & Weyman, A. Optimising workplace interventions for health and well-being. A commentary on the limitations of the public health perspective within the workplace health arena. International journal of workplace health management. 2013; 6(2) pp.104-117.

10. Weyman, A.K., Roy, D. and Nolan, P. One-way pendulum? Staff retention in the NHS: determining the relative salience of recognised drivers of early exit. International journal of workplace health management 2020; 13(1) pp.45-60.

11. Shen, J., Cox, A., & McBride, A. Factors influencing turnover and retention of midwives and consultants: a literature review. Health services management research. 2004; https://journals.sagepub.com/doi/abs/10.1258/0951484042317769 Retrieved 4/9/22

12. Fleming, G., & Taylor, B. J. An Evaluation of Day Support: A Community Rehabilitation Service? International Journal of Disability, Community & Rehabilitation, 2010; 9(1).

13. Bimpong, K.A.A., Khan, A., Slightm, R., Tolley, C.L. and Slight, S.P. Relationship between labour force satisfaction, wages and retention within the UK National Health Service: a systematic review of the literature BMJ Open 2020; 10(7). doi.org/10.1136/bmjopen-2019-034919

14. McVicar, A, Scoping the common antecedents of job stress and job satisfaction for nurses (2000–2013) using the job demands–resources model of stress. Journal of nursing management. Journal of Nursing Management 2016; 24(2); pp. E112-E136. Wiley Online Library. https://doi.org/10.1111/jonm.12326

15. Fletcher, L., Carter, M. and Lyubovnikova, J. Congruency of resources and demands and their effects on staff turnover within the English health care sector. Journal of Occupational and Organisational Psychology 2018; 91(3); pp. 688-696. Wiley Online Library. https://doi.org/10.1111/joop.12214

16. Sizmur S, Raleigh V. The risks to care quality and staff wellbeing of an NHS system under pressure. The King's Fund: Oxford. 2018 Jan 31:2

For peer review only

17. Lopez, V., Anderson, J., West, S and Cleary M. Does the COVID-19 Pandemic Further Impact Nursing Shortages? Issues in Mental Health Nursing, 2022; 43:3, 293-295, DOI: 10.1080/01612840.2021.1977875

18. Wilson C. Why stay in nursing? Nursing Management. 2006 Feb 1;12(9); 29-32.

19. Osaro, E. and Chima, N, Challenges of a negative workload and implications on morale, productivity and quality of service delivered in NHS laboratories in England. Asian Pacific Journal of Biomedicine 2021; 4(6); pp.421-429.

20. Kelliher, C. and Parry, E. Change in healthcare: the impact on NHS managers. Journal of Organizational Change Management 2015; 28(4); pp. 591-602

21. Wise, J. Survey of UK doctors highlights blame culture within the NHS. British Medical Journal BMJ 2018;362:k4001 https://www.bmj.com/content/362/bmj.k4001.full

22. Tingle, J. Embedding a just and learning culture in the NHS. British Journal of Nursing 2019; 28(15) Published Online:8 Aug 2019 https://doi.org/10.12968/bjon.2019.28.15.1028

23. Weyman, A. K. & O'Hara, R. Decision making at the front-line: the role of choice architecture in NHS paramedic judgements over patient conveyance Chapter 10 in: P. Wankhade (Ed) Critical Perspectives in Emergency Services Management. Routledge 2019.

24. Torjessen, I. NHS is "drowning in bureaucracy" and lacks good leaders, says scathing report. British Medical Journal 2021; Vol 351. doi.org/10.1136/bmj.h3901

25. Edwards N. Turning up the heat on the NHS. bmj. 2021; Oct 29;375.

26. Ravalier JM, McVicar A, Boichat C. Work Stress in NHS Employees: A Mixed-Methods Study. International Journal of Environmental Research and Public Health 2020; 17(18):6464. https://doi.org/10.3390/ijerph17186464

27. Rattray1, J.; McCallum, L,; Hul, A.; Ramsay, P.; Salisbury, P.; Scott, T,; Cole,S,; Miller, J.; and Dixon, D. Work-related stress: the impact of COVID-19 on critical care and redeployed nurses: a mixed-methods study. BMJ Open 2021; 11(7); pp.1-7.

28. Gulland, A. Many happy returns? Health Service Journal 2001; 111, 5776, pp.12-13

29. Newman, K., Maylor, U. and Chansakar, B. The nurse satisfaction, service quality and nurse retention chain": Implications for management of recruitment and retention. Journal of Management in Medicine 2022; 16(4) pp271-291.

30. Frijters, P., Shields, M., & Wheatley Price, S. Investigating the quitting decision of nurses: Panel data evidence from the British National Health service. Health Economics 2006; August. Retrieved from https://onlinelibrary.wiley.com/doi/abs/10.1002/hec.1144

For peer review only

31. Arnold, John; Loan-Clarke, John; Coombs, Crispin; Bosley, Sara; Martin, Caroline (2006): Push and pull: why allied health professionals stay in, leave or return to the NHS - views of professionals inside and outside the NHS. Loughborough University. Report. 2006; https://hdl.handle.net/2134/2364

32. Johnson, N., & Mandredi, S. Older Workers in the nursing and midwifery profession. Will they stay or will they go? Challenges of active aging. S. Manfreidi and L. Vickers (Eds) 2016; pp 139-158. Springer; Palgrave Macmillan, London.

33. Williamson, K and Williams, K.J. Organisational justice, trust and perceptions of fairness in the implementation of agenda for change. Radiography, 2011; 17(1), pp.61-66. https://doi.org/10.1016/j.radi.2010.08.004

34. British Medical Association (BMA). Stay one step ahead of the 2015 NHSPS. 2016. https://www.bma.org.uk/advice/employment/pensions/2015-nhs-pension-scheme. Retrieved 14/9/22

35. French, L., Hanna, P., & Huckle, C. “If I die, they do not care”: U.K. National Health Service staff experiences of betrayal-based moral injury during COVID-19. Psychological Trauma: Theory, Research, Practice, and Policy 2022; *14*(3), 516–521. https://doi.org/10.1037/tra0001134

36. Dean, E. Staff discontent rises as inflation continues to devalue salaries. Nursing Standard 2011; 26(3) September.

37. Weyman. A. K., Meadows, P. & Buckingham, A. Extending working life audit of research relating to impacts on NHS employees. May. NHS Working Longer Review 2013. https://www.researchgate.net/profile/Pamela Meadows/publication/264933033_Extending_Working_Life_Audit_of_research_relating_to_impacts_on_NHS_Employees/links/53f 607060cf2888a74923fd1/Extending-Working-Life-Audit-of-research-relating-to-impacts-on-NHS-Employees.pdf Retrieved 1//9/22

38. Atkinson, C. and Hall, L. Flexible working and happiness in the NHS. Journal of Employee Relations 2011; 22(1); pp.88-105.

39. Health Education England Facing the Facts, Shaping the Future. A draft health and care workforce strategy for England to 2027, 2017; https://www.hee.nhs.uk/sites/default/files/documents/Facing%20the%20Facts%2C%20Shaping%20the%20Future%20%E2%80%93%20a%20draft%20health%20and%20care%20workforce%20strategy%20for%20England%20to%202027.pdf. Retrieved 28/8/22

40. Nightingale, J., Burton, M., Appleyard, R., Stevens, T. and Campbell, S. Retention of radiographers: A qualitative exploration of factors influencing decisions to leave or remain within the NHS. Radiography (London) 2021; 27(3); pp.795-802. doi: 10.1016/j.radi.2020.12.008. Epub 2021 Jan 8. PMID: 33423914.

41. NHS Employers. Improving staff retention - a guide of employers 2017. https://www.nhsemployers.org/news/2017/09/improving-staff-retention-a-guide-for-employers. Retrieved 1/9/22

For peer review only

42. Royal College of Nursing. Staff retention: guidance for employers. Nursing Standard 2018; 32, 25, 15-15. doi: 10.7748/ns.32.25.15.s11. https://journals.rcni.com/nursing-standard/staff-retention-guidance-for-employers-ns.32.25.15.s11 Retrieved 14/9/22

43. NHS Improvement A collection of practical resources to help NHS organisations to improve staff retention. 2018; https://improvement.nhs.uk/resources/improving-staff-retention. Retrieved 1/9/22

44. Coster J, O'Hara R, Glendinning R, Nolan P, Roy D, Weyman A. Impact of working through COVID-19 on ambulance staff resilience and intention to leave the NHS: a mixed methods study Emergency Medicine Journal 2022;39:e5. http://dx.doi.org/10.1136/emermed-2022-999.38

45. Weyman, A., O'Hara R., Glendinning R., Nolan P., Roy D., Coster J. Impacts of working through COVID-19 pandemic on NHS staff and future employment intentions. Presented at HSR UK Conference 2022, Sheffield.

46. Thurstone, L. L. The Measurement of Values, Chicago, IL: University of Chicago Press, 1959.

47. Bock, R. D., & Jones, L. V. The Measurement and Prediction of Judgement and Choice, London, Holden-Day, 1968.

48. Cromer, M. K., Seaver, D. A., Stillwell, W. G., & Gaddy, C. D. Generating human reliability estimates using expert judgement, Vol.2, Maryland: The Maxima Corp. U.S. Nuclear Regulatory Commission, Contract Report, 1984; NUREG/CR - 3688/2.

49. Johnson, J., Hall, L., Berzins, K., Baker, J., Melling, K., & Thompson, C. Mental healthcare staff well-being and burnout: A narrative review of trends, causes, implications and recommendations for future interventions. International journal of mental health nursing 2018; 27, pp.20-32.

50. Kim, M. & Windsor, C. Resilience and work-life balance in first-line nurse manager. Asian nursing research, 2015; 9(1); pp.21-27.

51. Wicker, A. W. (1969). Attitudes versus actions: The relationship of verbal and overt behavioral responses to attitude objects. *Journal of Social Issues, 25*(4), 41–78. https://doi.org/10.1111/j.1540-4560.1969.tb00619.x

52. Barth, J., Greene, J.A., Goldstein, J. and Sibley, A. Adverse Health Effects Related to Shift Work Patterns and Work Schedule Tolerance in Emergency Medical Services Personnel: A Scoping Review. Cureus 2022; 1;14(4):e23730. doi: 10.7759/cureus.23730. PMID: 35509733; PMCID: PMC9060748.

53. Quaile, A. Tackling the shortage of paramedics. Journal of paramedic practice, 2015; 4(7) April DOI 10.12968/jpar.2015.7.4.167

For peer review only

54. Roberts, L. Long Covid worsens staff shortages in ambulance services The Telegraph 2022; 5th January. https://www.telegraph.co.uk/news/2022/01/05/long-covid-worsens-staff-shortages-ambulance-services/ Retrieved12/10/22

55. Gordon, S. Ambulance pressures taking a significant toll on staff, says UNISON, 2022; 25th April. https://www.unison.org.uk/news/press-release/2022/04/ambulance-pressures-taking-a-significant-toll-on-staff-says-unison/ Retrieved 12/10/22

56. Gregory, A. Thousands of NHS workers may quit for better-paid jobs, ministers warned. The Guardian 2022; 23rd May. https://www.theguardian.com/society/2022/may/23/thousands-of-nhs-workers-may-quit-for-better-paid-jobs-ministers-warned Retrieved 12/10/22

57. Morris, L. Four in five NHS staff ready to quit over pay. National Health Executive 2022; 17th June. https://www.nationalhealthexecutive.com/articles/four-in-five-workers-ready-to-quit-over-low-pay-nhs-workforce. Retrieved 10/9/22.

58. Buchan, J., Seccombe, I., Gershlick, B., & Charlesworth, A. In short supply. Pay policy and nurse numbers. The Health Foundation 2017.

59. Black, C. (2008) Working for a healthier tomorrow. London TSO.

60. Health and Safety Executive (2021) Work-related stress and how to manage it. https://www.hse.gov.uk/stress/causes.htm. Accessed 8/9/22.

61. Rajae-Joordens, R. and Engel, J. (2005) Paired comparisons in visual perception studies using small sample sizes. Displays, 26(1), 1-7. https://www.science direct.com/science/article/pii/SO14183820400708

For peer review only

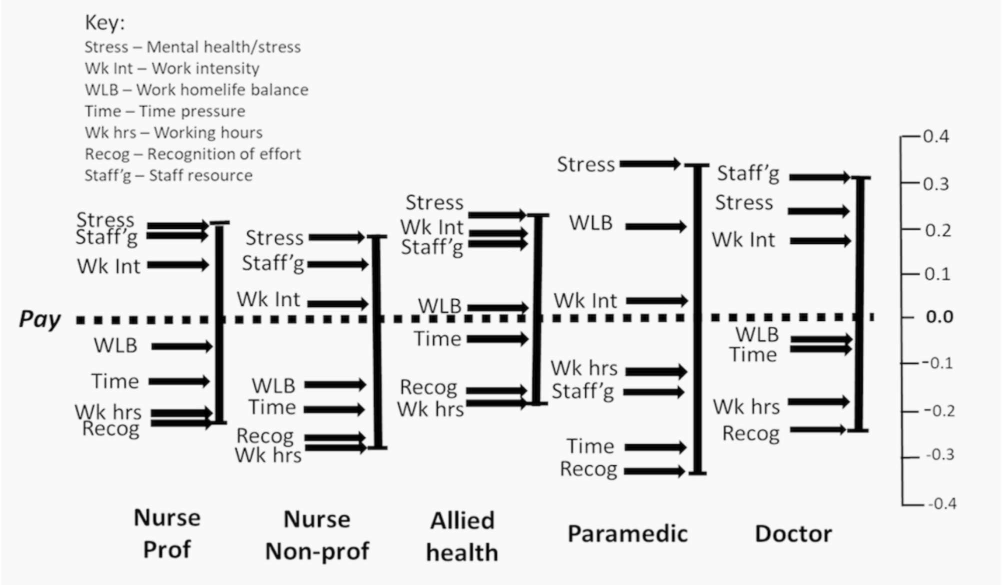

Figure 1 Weightings of push variables referenced to pay, by profession

707x413mm (72 x 72 DPI)

Appendix 1

Details of Push Variables ranking question

With refence to perceptions and beliefs relating to colleague behaviour, for a common set of variables (listed below).

Under the heading *'Why Do Staff Leave the NHS?' participants were presented with request::*

'We would like your views in more detail on how important the following issues are to explain why (RESPOPNDENT'S OCCUPATIONAL GROUP AUTOMATICALLY INSERTED HERE) staff leave the NHS. To make the task easier you will be presented with the issues two at a time**.'**

When each pair is presented, choose the issue that you think is the more important reason for (RESPOPNDENT'S OCCUPATIONAL GROUP AUTOMATICALLY INSERTED HERE) staff leaving the NHS.

Item set:

A. Time pressure
B. Staffing levels
C. Working hours
D. Workload (intensity of work)
E. Work/home-life balance
F. Pay
G. Mental health/stress
H. Recognition of contribution

Permutations of pairings (*N* = 28)

A&B; A&C; A&D; A&E; A&F; A&G; A&H

B&C; B&D; B&E; B&F; B&G; B&H

C&D; C&E; C&F; C&G; C&H

D&E; D&F; D&G; D&H

E&F; E&G; E&H

F&G; F&H

G&H

The data was gathered using an on-line platform hosted by YouGov. To mitigate presentation order bias, the order of pairings, as well as the order within each pairing (i.e. A&B or B&A) was randomised.

For peer review only

For peer review only

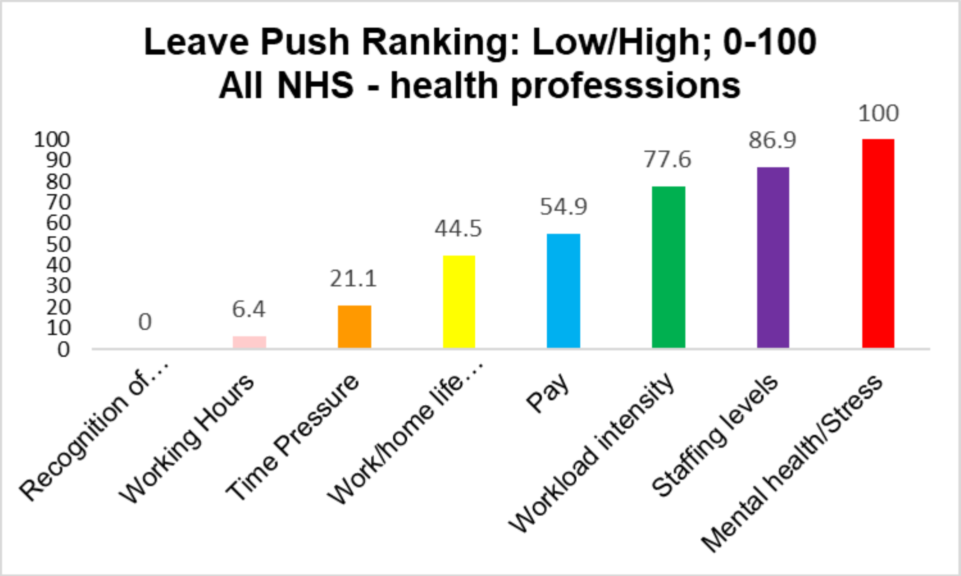

Relative salience of push variables – all NHS Health Professions

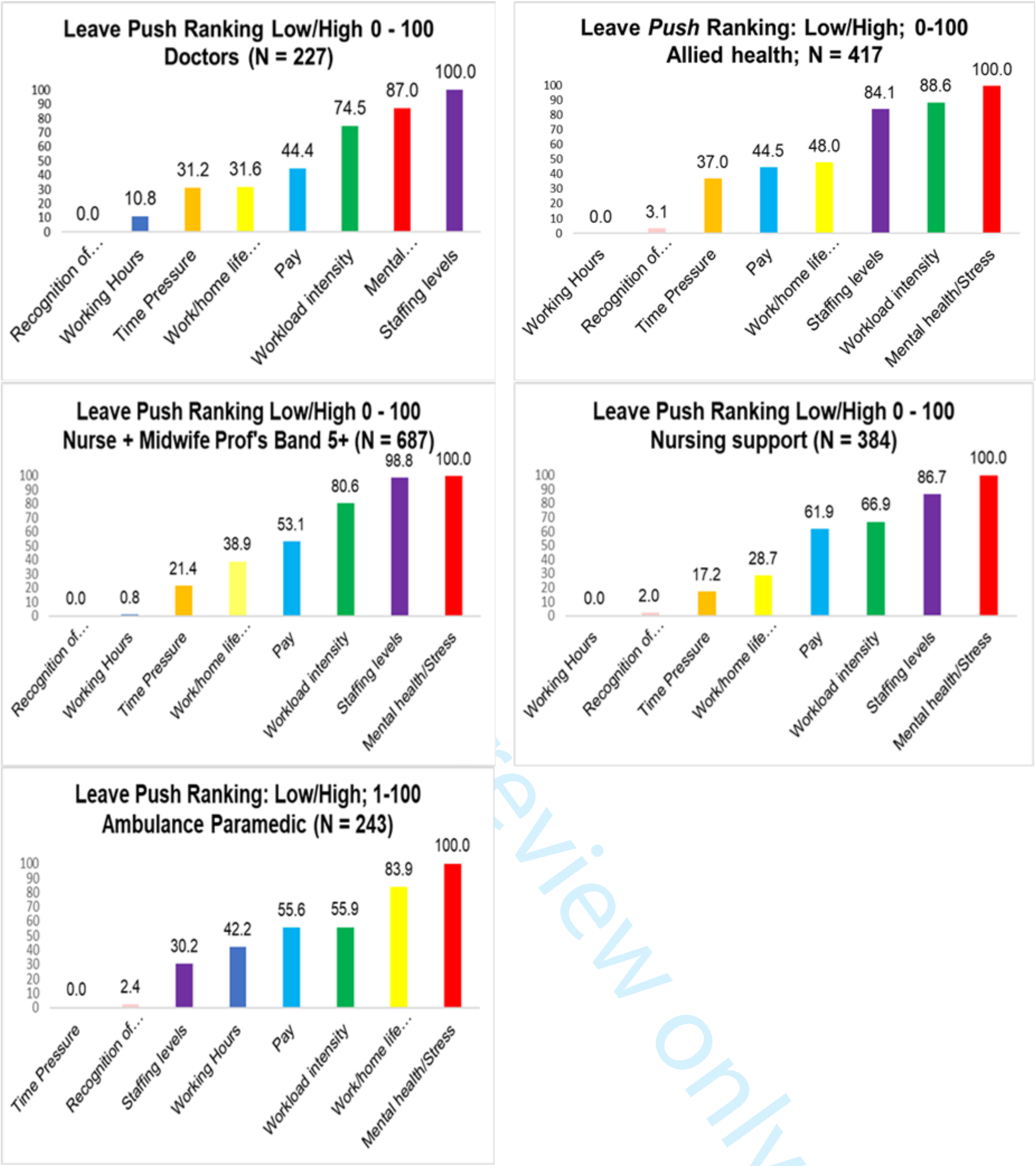

Relative salience of push variables – by Profession job-family

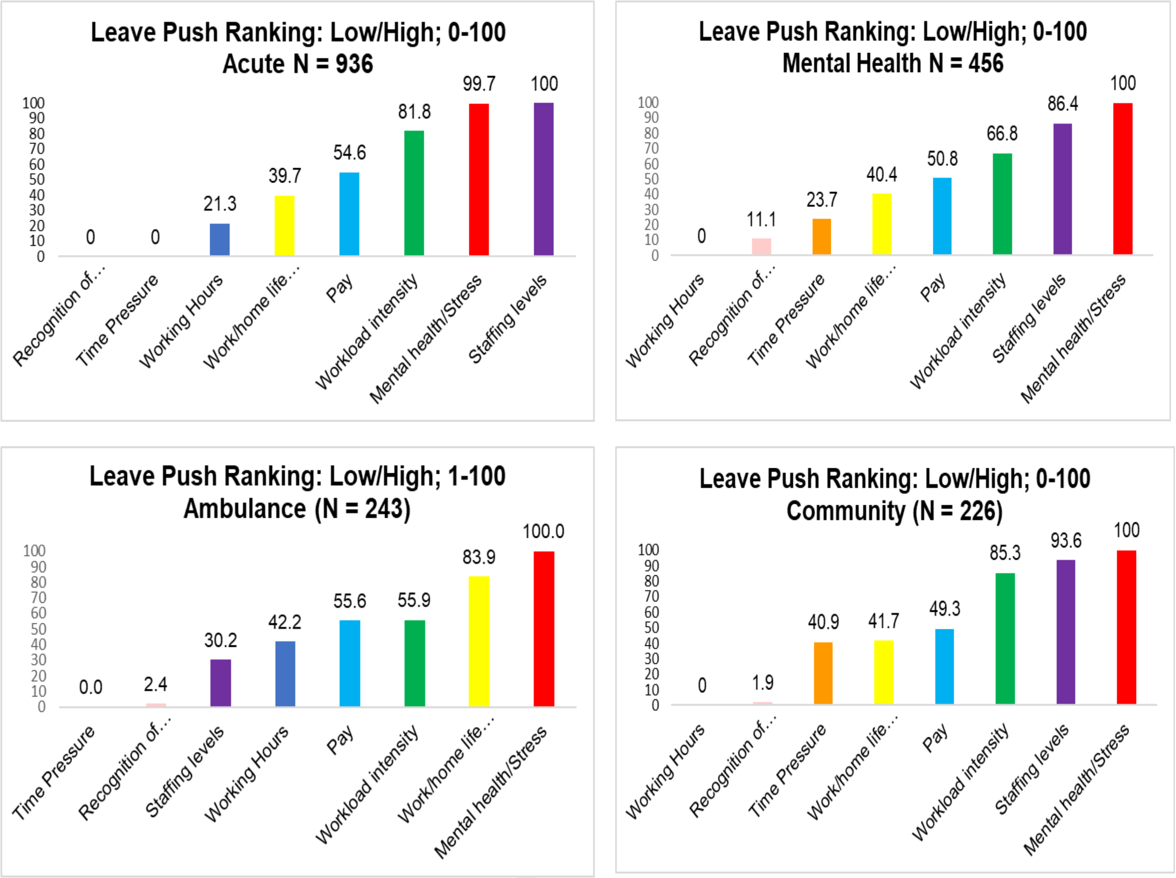

Relative salience of push variables – by care-provider organisation type

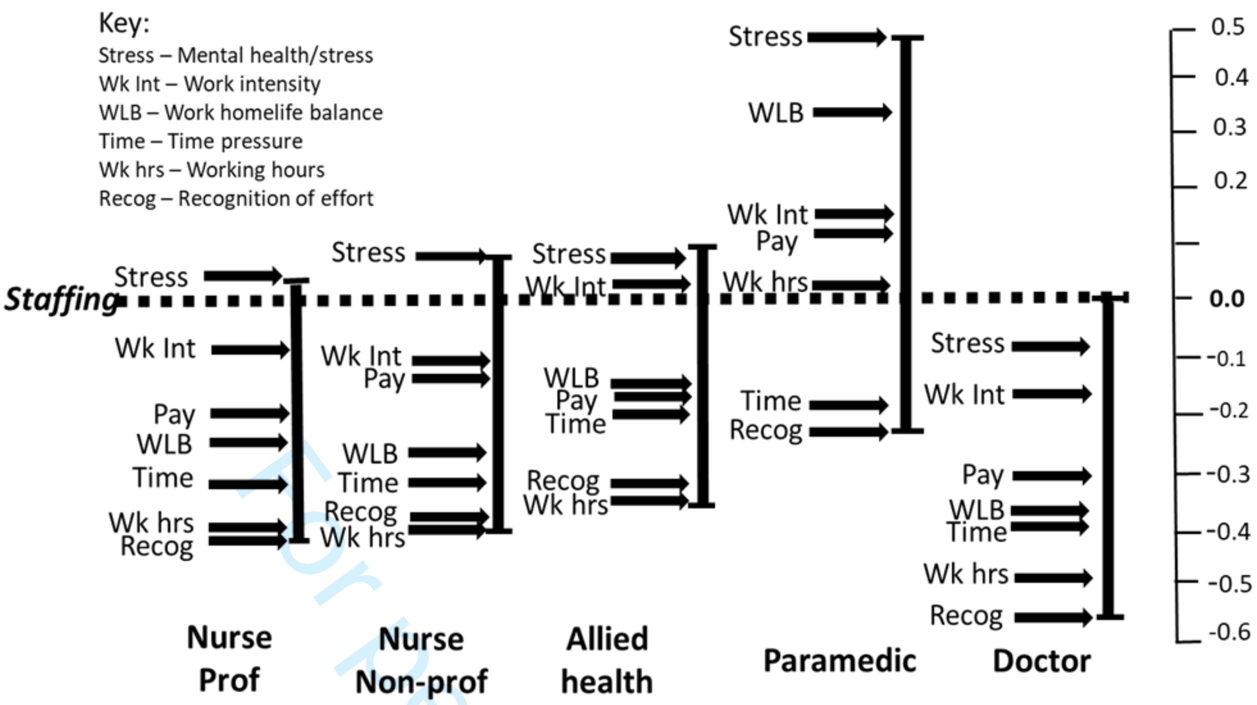

Weightings of *push* variables referenced to staffing resource level, by profession

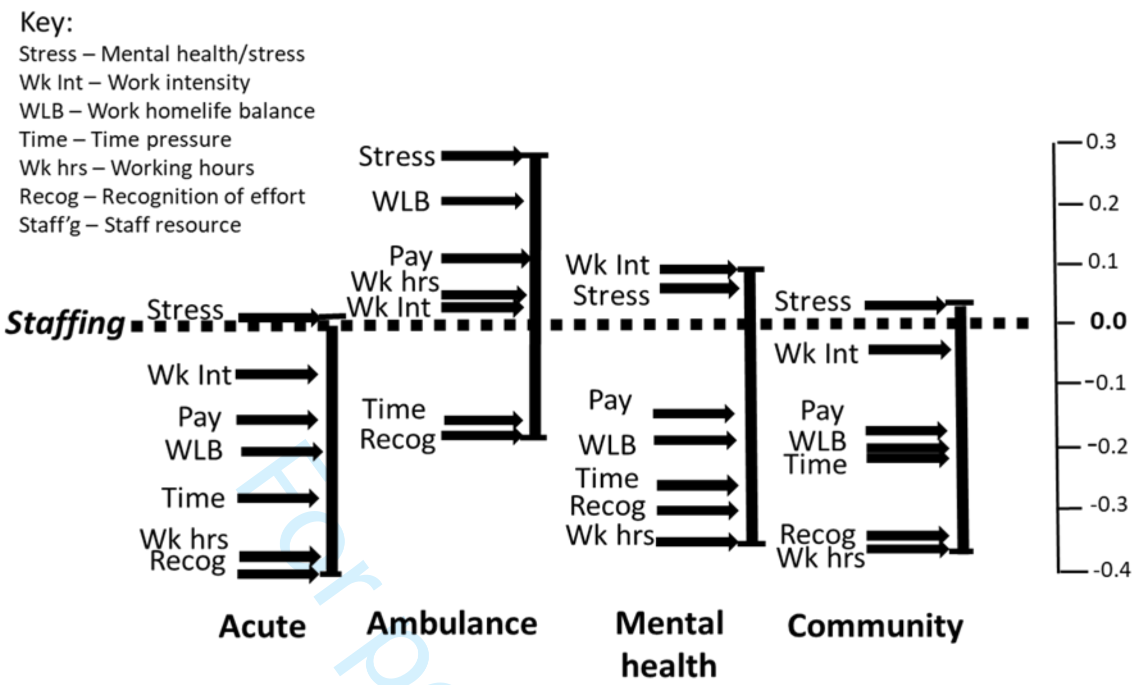

Weightings of *push* variables referenced to staffing resource level, by care provider organisation type
